# Dietary fructo-oligosaccharides dose-dependently modulate the microbiome and suppress type 2 lung inflammation in a murine model of house dust mite-induced allergic asthma

**DOI:** 10.3389/fnut.2026.1705988

**Published:** 2026-03-23

**Authors:** Roos E. M. Verstegen, Marjolein J. W. de Bruijn, Johan Garssen, Gert Folkerts, Atanaska I. Kostadinova, Rudi W. Hendriks, Linette E. M. Willemsen

**Affiliations:** 1 Division of Pharmacology, Faculty of Science, Utrecht Institute for Pharmaceutical Sciences, Utrecht University, Utrecht, Netherlands; 2 Department of Pulmonary Medicine, Erasmus Medical Centre (MC), University Medical Centre Rotterdam, Rotterdam, Netherlands; 3 Danone Research and Innovation, Utrecht, Netherlands

**Keywords:** allergic airway inflammation, fructo-oligosaccharides, microbiome, short-chain fatty acids, type 2 inflammation

## Abstract

**Introduction:**

A balanced microbiome is crucial for local and systemic immune regulation. Dietary fibers can support the intestinal microbiome, protecting the host from allergic diseases, including asthma. The effects of fibers depend on their type, dose, and disease context. Here, we investigated the preventative effects of four doses of fructooligosaccharides (FOS) in a murine model for house dust mite (HDM)-induced allergic asthma.

**Methods:**

BALB/c mice received a diet containing 1%, 2.5%, 5%, or 10% FOS (w/w) both prior to and during sensitization and challenges with HDM. Bronchoalveolar lavage fluid (BALF), lung tissue, serum, and cecum content were collected at the endpoint. Fecal microbiome composition was analyzed, and levels of short-chain fatty acids (SCFAs) were measured in cecum content, serum, and lung samples.

**Results:**

HDM-allergic mice showed eosinophilic airway inflammation and increased pulmonary type 2 inflammation, while cecal SCFA levels were lower compared to sham mice. Serum acetate concentrations showed a similar decline (*p* = 0.092). The 10% FOS diet did not prevent allergic sensitization or eosinophilic airway inflammation; however, it significantly reduced the proportions of T helper 2 (Th2) cells and the Th2/Th1 ratio in the lungs, decreased concentrations of chemokine (C-C motif) ligand 2 (CCL22) and interleukin (IL-13) in the BALF, and inhibited IL-13 production upon *ex vivo* HDM restimulation of lung cells. The 2.5% and 5% FOS diets also decreased Th2 cell frequency in the lungs. High doses of FOS increased the abundance of fecal *Prevotellaceae*, while reducing fecal *Oscillospiraceae* and *Lactobacillaceae*. These microbial shifts were correlated with protective effects against type 2 inflammation. In HDM-allergic mice, fecal *Prevotellaceae* abundance correlated positively with serum acetate concentrations, which were correlated with type protective effects. In allergic mice, the 2.5% and 5% FOS doses were associated with increased abundance of fecal *Muribaculaceae* and *Bacteroidaceae*, respectively, along with elevated cecal SCFA concentrations. In addition, the 5% FOS dose increased the relative abundance of fecal *Lachnospiraceae*, which correlated negatively with serum acetate levels and type prevention.

**Discussion:**

Dietary FOS modulated the gut microbiome and attenuated pulmonary type 2 immune responses in a dose-dependent manner. These findings underscore the importance of fiber dosing for precision nutrition strategies in allergy management.

## Introduction

Asthma is a global health problem, affecting an estimated 300 million people worldwide (1). Allergic asthma, considered a sub-category of asthma, is characterized by type 2 immune responses and can be induced by environmental allergens such as house dust mite (HDM) (2). The increased prevalence of asthma and other allergic diseases is often explained by the “biodiversity hypothesis” (3, 4), which proposes that lifestyle and environmental changes can lead to misdirected immune responses to allergens (4). Environmental pollutants, dysfunction of epithelial barriers, oxidative stress, and low-grade inflammation can affect immunological pathways that promote type 2 inflammation, and consequently, the development of allergic diseases (5). The role of the intestinal microbiome in immune regulation and asthma development has received growing attention (6).

Although asthma management has become more precise and targeted (7), current asthma treatments focus on controlling the disease and relieving patients' symptoms (8) rather than curing the disease. Given the high prevalence of asthma and no existing cure, there is great interest in disease prevention. Targeting the gut microbiome could be a promising strategy.

There is no general definition of a healthy gut microbiome (9). In addition to greater species diversity and a better balance between species (10), the functional capacity, stability, resilience, and the specific host context play a role in this concept (9). Interventions promoting this diversity and balance include probiotics, prebiotics, and synbiotics (11). Prebiotics, most often dietary fibers, are fermented by bacteria in the gut, resulting in the production of, among others, the short-chain fatty acids (SCFAs) acetate, propionate, and butyrate. SCFAs can modulate the systemic immune system and regulate epithelial barrier function via several mechanisms. These include G-protein-coupled receptor binding or modification of gene expression at the epigenetic level by affecting histone deacetylase activity (12). SCFAs may also serve as ligands for the aryl hydrocarbon receptor (AhR) (13) or as metabolic substrates (e.g., in the tricarboxylic acid cycle) (14).

A higher fiber intake does not necessarily lead to higher intestinal SCFA levels. In a study in rats, 5–10% (w/w) inulin in the diet increased cecal SCFA levels more than 20% inulin (15). Given the variety of effects that dietary fibers have on both the microbiome and SCFA production, and consequently on immune regulation, optimization of the dosing of specific dietary fibers in a specific disease context is essential.

Previous studies investigating a combination of 1% (w/w) fructo-oligosaccharides (FOS) (1:1) with *Bifidobacterium* breve, or 1% (w/w) galacto-oligosaccharides (GOS), showed prevention of both eosinophilic airway inflammation and type 2 inflammation in an acute HDM-induced murine asthma model (16–18). Others have shown that dietary supplementation with doses of dietary fibers higher than 1% resulted in pronounced effects (19–21). In the present study, we investigated the effects of dietary supplementation with different doses (1, 2.5, 5, and 10%) of FOS (1:1 short-chain:long-chain ratio) on the development of acute HDM-induced allergic airway inflammation and on the modification of microbiome composition and function in a mouse model.

## Materials and methods

### Diets

The basic diet was a semi-synthetic AIN93G diet without glucose or lactose, with 15% (w/w) cellulose instead of 5% (to allow interexchange with FOS), with soy protein instead of casein, and with methionine at the expense of cysteine (ssniff-Spezialdiëten GMBH, Germany), in line with a previous study from our group (16–18). In the experimental diets, cellulose was substituted with different percentages [1, 2.5, 5, or 10% [w/w]] of sc-FOS (Raftilose P95, Beneo, Belgium) and lc-FOS (inulin HP, Cosucra Warcoing, Belgium) in a 1:1 mixture. Details of the dietary composition are provided in Supplementary Table S1. Access to food and sterile water was *ad libitum*.

### Animals

A total of 52 male BALB/cAnNCrl mice (Charles River, Germany), aged 7 weeks, were housed in individually ventilated cages. Housing conditions were as previously reported (22). Upon arrival, mice were randomly assigned to cages and to one of the seven experimental groups (more information in the next section) using Microsoft Excel for randomization (four mice per cage of the same experimental group). Dietary intervention started upon arrival at the animal facility. After 2 weeks of acclimatization, including the dietary intervention, the HDM sensitization and challenge protocol was initiated (described in the next section). This study was conducted under an ethical license provided by the national competent authority (CCD, Centrale Commissie Dierproeven; AVD1080020198826) following positive advice from the Ethical Committee on the Use of Laboratory Animals of Utrecht University. All animal procedures were captured in protocols approved by the Animal Welfare Body, in accordance with the institutional Guidelines of the Ethical Committee of Utrecht University, thereby ensuring full compliance with the European Directive 2010/63/EU for the use of animals for scientific purposes.

Some outcome parameters of the sham and allergic mice fed a control diet will be reported in another publication (22).

### Animal procedures

In line with previous studies (17, 22), an allergic airway inflammation model was performed. On day 0, mice were sensitized via intranasal (i.n.) administration of 40 μL phosphate-buffered saline (PBS) ± 5 μg HDM (02.01.86, Citeq, The Netherlands), under isoflurane anesthesia (induction: 4% O_2_; maintenance: 1.5% O_2_). On days 7–11, mice were challenged intranasally with 40 μL PBS ± 15 μg HDM. Feces were collected on day 11 by placing individual mice in a box for 2 min. Body weight and food intake were measured weekly. On day 14, mice were anesthetized via intraperitoneal injection of 4.92 mg ketamine (Narketan^®^10, Vetoquinol, France) per 25 g mouse and 0.033 mg Dexdormitor (Dexdormitor^®^, Vetoquinol, Orion Pharma, Espoo, Finland) per 25 g mouse in a total injection volume of 150 μL sterile saline. Adequate anesthesia was confirmed by the absence of reflex responses before subsequent procedures. Terminal blood collection was performed via cardiac puncture under deep anesthesia, and animals were euthanized by exsanguination. Bronchoalveolar lavage fluid (BALF), lung tissue, and cecal contents were also collected. An overview of the experimental groups is provided in Table 1.

**Table 1:** Overview of experimental groups.

**Group**	**Diet**	**Treatment sensitization/challenge**	**Number of animals**
Sham	AIN93G (control diet)	PBS/PBS	4
Sham FOS 5%	AIN93G + 5% FOS	PBS/PBS	8
Allergic (+HDM)	AIN93G (control diet)	PBS+5 μg HDM/PBS+15 μg HDM	8
FOS 1% (+HDM)	AIN93G + 1% FOS	PBS+5 μg HDM/PBS+15 μg HDM	8
FOS 2.5% (+HDM)	AIN93G + 2.5% FOS	PBS+5 μg HDM/PBS+15 μg HDM	8
FOS 5% (+HDM)	AIN93G + 5% FOS	PBS+5 μg HDM/PBS+15 μg HDM	8
FOS 10% (+HDM)	AIN93G + 10% FOS	PBS+5 μg HDM/PBS+15 μg HDM	8

#### Bronchoalveolar lavage fluid (BALF) collection and lung cell suspension preparation

BALF collection and preparation of lung cell suspensions were conducted as previously described (17). Briefly, mouse lungs were flushed with 1 mL of 0.9% NaCl (37°C) containing protease inhibitor (Roche, Switzerland) and 3 x 1 mL 0.9% NaCl (37°C). The final three lavages were pooled, and all lavages were centrifuged (400*g*, 5min). The supernatant of the first mL was stored at −20 °C until further analysis. The supernatant of the pooled lavages was discarded. The cell fractions from the first mL and the pooled lavages were combined and resuspended in 150 μL of 0.9% NaCl. Total BALF cells were counted using a Bürker-Türk chamber (100 × ).

Lungs were digested at 37 °C with DNase I (Roche) and Collagenase A (Roche). After 30 min, digestion was stopped with fetal calf serum (FCS) (Hyclone Laboratories, USA). Tissue was filtered (70 μm), rinsed with RPMI 1640 (Lonza, USA), and centrifuged (1,400 rpm, 5 min, 4 °C). Red blood cells were lysed with buffer [dH_2_O + 8.3 g/L NH_4_Cl + 1.0 g/L KHCO_3_ + 37.2 mg/L ethylenediaminetetraacetic acid 9EDTA)] for 4 min, then neutralized with restimulation medium (RPMI 1640 culture medium + 10% fetal calf serum (FCS) + 1% penicillin-streptomycin solution (Sigma-Aldrich, USA) + 0.02% 2-mercaptoethanol]. After a final centrifugation, cells were resuspended in restimulation medium.

#### Flow cytometry

Flow cytometric analyses of BALF and lung cells were conducted. Briefly, 60 μL of the pooled BALF cell suspension or 1 × 10^6^ lung cells were added to each well. Cells were stained with fixable viability dye (eFluor 780, Thermo Fisher Scientific, USA), followed by incubation with block buffer [PBS with 1% BSA, 5% FCS, and 1% cluster of differentiation 16 (CD16)/CD32 block solution [Thermo Fisher Scientific]] to prevent non-specific antibody binding. A complete overview of the antibodies used, including corresponding isotype controls, is presented in Supplementary material.

Antibody panels included:

Panel 1—BALF cells: CD3e-PE-Cy7, MHCII-FITC, CD11c-APC, B220-Pe Cy7, and CCR3-PE in FACS buffer (1% FCS in PBS).

Panel 2—Lung cells: CD4-BV510, CD69-PE-Cy7, CXCR3-PE, and T1ST2-FITC.

Panel 3—Lung cells: CD4-BV510, CD25-PerCP-Cy5.5, CD127-PE-Cy7, and CD196-PE.

Lung cells were fixed and permeabilized using the Foxp3 Transcription Factor Staining Buffer Set (eBioscience, USA) and exposed to FoxP3-FITC and Rorγt-APC (Panel 2) the following day. Flow cytometric measurements of BALF cells were performed using a FACS Canto II (BD Biosciences, Erembodegem, Belgium). Measurements of lung cells were performed using a CytoFLEX (Beckman Coulter, USA). Data were analyzed using FlowLogic Software version 1.0 (Inivai Technologies, Australia).

For quantification of group 2 innate lymphoid cells (ILC2) and cytokine production, BALF cells (70 μL of pooled resuspension) were centrifuged (400 g, 7 min). The pellet was incubated in RPMI 1640 (Gibco, Waltham, MA, USA) + 10% FCS + Phorbol 12-myristate 13-acetate (PMA) (Merck, Germany) + Ionomycin (Merck) and Golgistop (BD Biosciences) for 4 h (37 °C and 5% CO_2_). Then, cells were washed with MACS buffer (Homemade) and exposed to Fc-block and extracellular antibodies (including antibodies for gating lineage-negative cells):

Panel 4—BALF cells: CD11b-PE, CD11c-PE, CD19-PE, B220-PE, NK1.1-PE, GR-1-PE, Ter119-PE, FceRIa-PE, CD4-FITC, CD3-PE Texas Red, CD25-Pe Cy7, Sca-1 – BV786, CD90.2-AF700 and CD8-APC eFluor780.

After 30 min (4 °C), cells were washed with PBS and incubated for 15 min with Fixable Viability Dye. The cells were then washed with PBS, fixed in 2% PFA, and permeabilized with 0.05% Saponin (Sigma-Aldrich). Subsequently, cells were stained with antibodies against IL-9-PerCP Cy5.5, IL-13-EF450, interferon-gamma (IFN-γ)-BV650, IL-4-BV711, and IL-5-allophycocyanin (IL-5-APC) for 1 h at 4 °C. Measurements of BALF ILC2s were performed using a FACS Symphony A5 (BD Biosciences), and data were analyzed using FlowJo version 10 (BD Biosciences).

#### Ex vivo HDM-restimulation of lung cells

Lung cell suspensions were *ex vivo* restimulated with HDM, as described in previous research (22). Briefly, lung cells (4 × 10^5^ per well) were cultured with or without 25 μg/mL HDM (Citeq) for 6 days. Supernatants were collected for cytokine analysis, and cell metabolic activity was assessed using a WST-1 assay (Roche).

#### Cecal content, serum, and lung homogenate preparation

Cecum content, serum, and lungs were processed for further analyses using previously described protocols (22). Briefly, cecal content samples were homogenized and centrifuged. Serum was collected via cardiac puncture from anesthetized mice and centrifuged. Lung tissues were homogenized in PBS with Triton X-100 and protease inhibitors (Roche, Basel, Switserland), then centrifuged. Cecum homogenate supernatant, serum fraction, and lung homogenate supernatant were stored at −20 °C. For lung homogenates, protein concentrations were standardized based on protein content determined using the Pierce BCA Protein Kit (Thermo Fisher Scientific).

#### Chemokine, cytokine, and antibody measurements in serum, BALF, and ex vivo restimulation supernatant using ELISA

Protein concentrations in different tissue samples were analyzed as described previously (22). Briefly, serum HDM-specific IgE and total IgE were determined using an in-house developed and validated protocol (23). HDM (Citeq) and 1 μg/mL rat anti-mouse IgE (BD Pharmingen, USA) were used as capture antibodies, and 1 μg/mL biotin anti-mouse IgE (BD Pharmingen) was used as the detection antibody.

BALF concentrations of IL-5, IL-13 (Thermo Fisher Scientific), and CCL22 (R&D Systems, USA), as well as *ex vivo* restimulation supernatant concentrations of IL-13 and IL-10 (Thermo Fisher Scientific), were measured according to the manufacturer's protocol. Final readouts were performed using GloMax Discover (Promega, USA). HDM-specific IgE levels are expressed as optical density (OD at 490 nm), and all samples were measured in the same ELISA plate and at the same dilution.

#### SCFA quantification

As described in more detail previously (22), acetate, propionate, and butyrate levels of cecal content supernatants, serum, and lung tissue homogenate supernatants were determined using 10 μL of sample by LC-MS/MS (Nexera X2, Shimadzu; QTRAP^®^ 5500, AB SCIEX), using the following internal standards: acetic acid-d4 (50 μM) (Thermo Fisher Scientific), propionic acid-d3 (20 μM) (Toronto Research Chemicals, Canada), and butyric acid-d7 (10 μM) (Cayman Chemical, USA). SCFA concentrations in cecum content and lung were normalized to tissue weight and expressed as mol/g.

#### Fecal microbiome analysis

Fecal samples were analyzed by BGI Genomics (Hong Kong) as previously described (22). Briefly, DNA was extracted using the MagPure DNA KF Kit B (MAGEN, Guangzhou, China). 16S ribosomal RNA (rRNA) gene sequencing was performed using the V3–V4 variable region of 16S rDNA with the following primers: 338F: ACTCCTACGGGAGGCAGCAG, 806R: GGACTACHVGGGTWTCTAAT. The DNBSEQ-G400 platform (BGI, Shenzhen, China) was used to generate sequencing reads.

Alpha diversity (Shannon's index) was calculated using Mothur (version 1.31.2). Beta diversity (weighted UniFrac) was analyzed using a permutational multivariate analysis of variance (PERMANOVA) test in QIIME (version 1.8.0). Taxonomy assignment was performed at the Operational Taxonomic Unit (OUT) level using the Greengene V202210 database with the RDP classifier (v2.2). Bacterial composition was analyzed at the family level.

### Statistics

G^*^Power 3.1.9.2 software was used to determine the sample size. This included an analysis of variance (ANOVA) (fixed effects, omnibus, and one-way) with *a priori* power analysis. The effect size was based on BALF inflammatory cell influx results from our previous study. One mouse was excluded from the allergic (control diet) group because it failed to develop increased inflammatory markers (values resembled those of the sham group).

GraphPad Prism (version 10.4.1) was used for statistical analysis. Data were tested for Gaussian distribution and transformed when necessary. Sham (control diet) vs. allergic (control diet) mice were compared to determine allergic airway inflammation development, and sham control vs. sham FOS 5% were compared to study the basal effects of FOS on immune and microbiome parameters. Statistical tests included the unpaired *t*-test, non-parametric Mann–Whitney test (for non-normally distributed data) or unpaired (two-tailed) *t*-test with Welch's correction (for heteroscedastic variance), depending on compliance with test conditions. Dietary effects on allergy were tested using one-way ANOVA with *post-hoc* Dunnett's multiple comparisons test, Kruskal–Wallis with *post-hoc* Dunn's multiple comparisons test (for non-normally distributed data), or Brown–Forsythe and Welch ANOVA test with *post-hoc* Dunnett's T3 multiple comparisons test (for heteroscedastic variance), depending on compliance with test conditions. For uniform presentation of all parameters, data are expressed as mean ± standard error of the mean (SEM), with individual measurements shown. Correlations were determined using computing non-parametric Spearman correlations. The correlation analysis did not include False Discovery Rate correction due to the exploratory nature of the study. *p* < 0.05 was considered statistically significant.

## Results

### Dietary intervention with FOS has limited effects on eosinophilic airway inflammation in HDM-allergic mice

**Figure 1:**
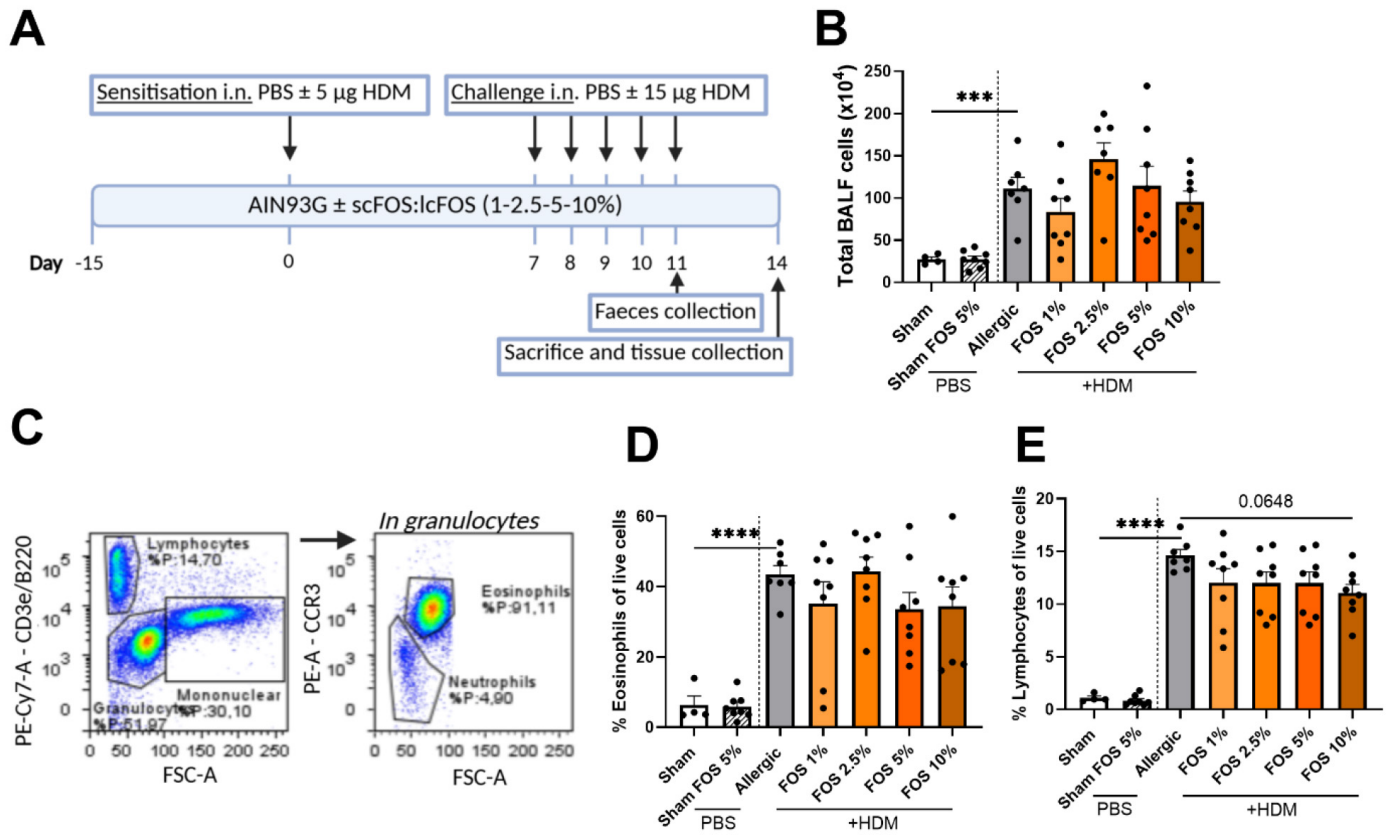
Effects of FOS on airway inflammation in a house dust mite (HDM)-induced acute asthma model. **(A)** Visual representation of the acute HDM-induced allergic airway inflammation model in mice. On day 15, BALB/c mice were received and assigned to an AIN93G diet with or without fructo-oligosaccharides (1, 2.5, 5, and 10%). Diets were sustained until the end of the study (day 14). Mice were intranasally (i.n.) sensitized on day 0 with PBS or PBS + 5 μg HDM. Mice were challenged i.n. on days 7–11 with PBS or PBS + 15 μg HDM. Feces were collected on day 11, and mice were sacrificed on day 14. **(B)** Inflammatory cell infiltration in the broncho-alveolar lavage fluid, as determined by counting with a Bürker–Türk chamber. **(C)** Representative gating strategy for flow cytometric analysis of cells obtained from BALF (eosinophils and lymphocytes). **(D)** Percentage of eosinophils and lymphocytes in BALF as determined by flow cytometry. The experimental model (sham vs. allergic) and sham 5% FOS effect comparisons were analyzed using an unpaired t-test [**(B)** and **(E)** sham–sham FOS 5%], **(D)** or Welch's t-test [**(B)** and **(E)** experimental model]. The effect of the diets on HDM allergy [allergic (HDM)-control diet vs. allergic (HDM)-FOS diet groups] was tested using one-way ANOVA followed by post-hoc Dunnett's multiple comparisons test. *N* = 7–8 (sham *N* = 4). Results are shown as mean ± SEM (***p < 0.001, ****p < 0.0001). Figure 1A was created using BioRender.

In an acute HDM-induced allergic airway inflammation model, mice received a dietary intervention containing FOS at four different doses [1, 2.5, 5, and 10% [w/w]] before and during sensitization and challenge with HDM (Figure 1A). Dietary intake and final body weight did not vary between the different intervention groups (Supplementary Figure S1). HDM exposure significantly increased total cell influx in the bronchoalveolar lavage fluid (BALF) (Figure 1B). Cell differentiation analyses (Figure 1C; see Supplementary Figure S2A for extended gating strategy) showed that the influx of BALF cells was mainly attributable to eosinophils and lymphocytes (Figures 1D, E), and not to neutrophils (Supplementary Figure S2B). Dietary interventions with FOS did not alter overall cell influx in the BALF or eosinophil numbers (Supplementary Figures 1B, D). The 10% FOS tended (*p* = 0.065) to decrease the percentage of lymphocytes compared to control-fed HDM-allergic mice (Supplementary Figure 1E). The increased percentage of ILC2 in the BALF, as well as increased serum HDM-specific IgE and total IgE in HDM-allergic mice, were not affected by FOS supplementation (Supplementary Figure S3).

### Dietary intervention with 2.5–10% (w/w) FOS decreases pulmonary Th2 cell frequency

**Figure 2:**
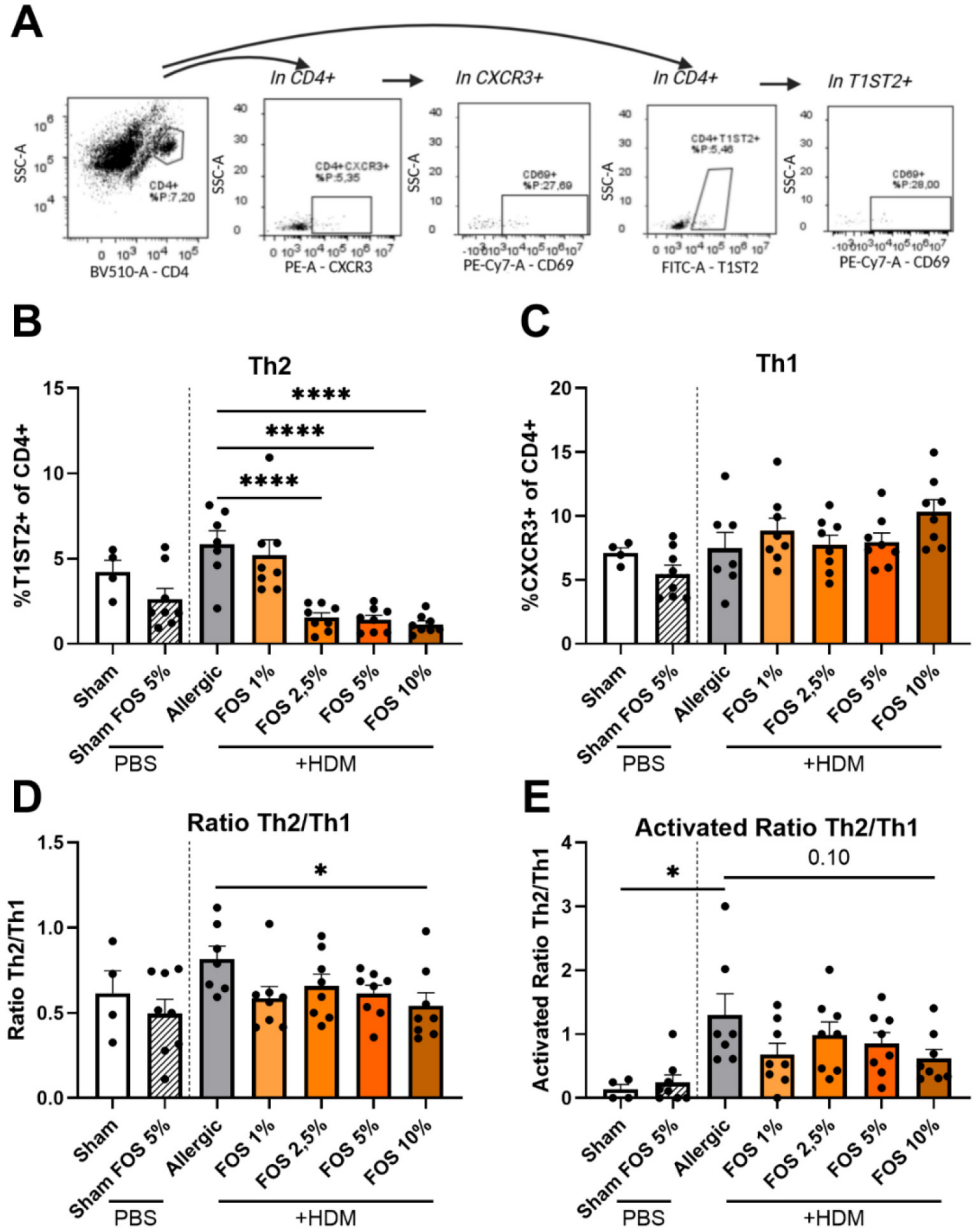
Effects of FOS on Th-cell phenotype in the lungs of HDM-allergic mice. **(A)** Representative gating strategy for flow cytometric analysis of lung cells. Cells were identified as: %T1ST2+ of CD4+ (Th2), %CXCR3+ of CD4+ (Th1), %T1ST2+CD69+ of CD4+ (activated Th2) and %CXCR3+CD69+ of CD4+ (activated Th1). Frequency of **(B)** Th2 cells, **(C)** Th1 cells, and **(D)** the ratio of Th2/Th1 cells in a lung cell suspension. **(E)** the ratio of activated Th2 vs. activated Th1 in lung cell suspension. The experimental model (sham vs. allergic), and sham vs. 5% FOS effect comparisons were analyzed using an unpaired t-test [**(B–D)**, and **(E)** Sham-Sham FOS 5%] or Welch's t-test [**(E)** experimental model]. The effect of the diets on HDM allergy [allergic (HDM)-control diet vs. allergic (HDM)-FOS diet groups] was tested using One-way ANOVA followed by post-hoc Dunnett's multiple comparisons test. *N* = 7–8 (sham *N* = 4). Results are shown as mean ± SEM (*p < 0.05, ****p < 0.0001).

Next, we examined the pulmonary T lymphocytes in more detail. We performed flow cytometric analyses on lung cells in suspension (Figure 2A; see Supplementary Figure S4A for extended gating strategy). Lung Th2-cell frequency, defined as the percentage of T1ST2+ cells within the CD4+ cell population, was significantly decreased by dietary intervention with FOS doses of 2.5, 5, and 10% (Figure 2B). Although the frequency of Th1 cells was not significantly affected by the diets (Figure 2C), the Th2/Th1 ratio shifted away from Th2 with the 10% FOS diet (Figure 2D). No significant effect on the activated Th2/Th1 ratio (defined as the CD69+ fraction of Th2 and Th1 cells; Supplementary Figures S4B, C) was observed, although the 10% FOS diet showed a trend (*p* = 0.10) toward shifting the activated Th2/Th1 ratio away from Th2 (Figure 2E). Collectively, these results indicate a progressive decline in the lymphocyte fraction within the BALF and a phenotypic shift of pulmonary lymphocytes away from the Th2 profile, particularly pronounced after administration of 10% FOS. Flow cytometric analyses of Th17 (CD4+CD196+Rorγ+) and Treg (CD4+CD25+CD127-Foxp3+) cells showed no effects of the diets on the cell frequency (Supplementary Figure S5).

**Figure 3:**
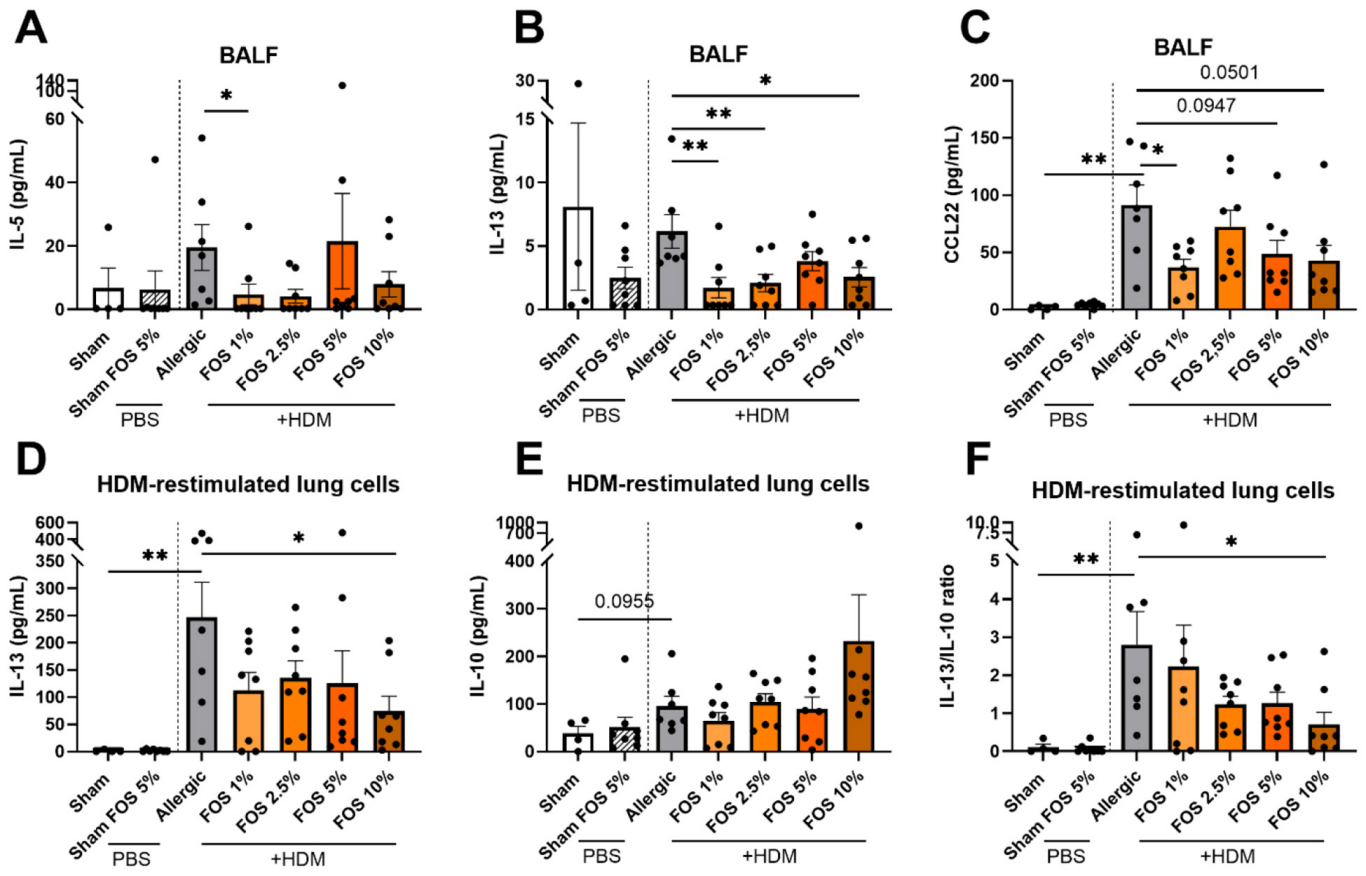
Effects of FOS on cytokine and chemokine production in the lung of HDM-allergic mice. Concentrations of **(A)** IL-5, **(B)** IL-13, and **(C)** CCL22 in the bronchoalveolar lavage fluid (BALF). Lung cells were isolated and ex vivo restimulated with HDM. Cytokine concentrations of **(D)** IL-13 and **(E)** IL-10 were measured in the supernatant of the cell culture after 6 days of incubation, and **(F)** their ratio was calculated. The experimental model (sham vs. allergic), and sham vs. 5% FOS comparisons were analyzed using an unpaired t-test [**(B, C)** Sham-Sham FOS 5%, **(D)** Sham-Sham FOS 5%, **(E, F)**], Mann–Whitney test **(A)** or Welch's t-test [**(C)** experimental model and **(D)** experimental model]. The effect of the diets on HDM allergy [allergic (HDM)-control diet vs. allergic (HDM)-FOS diet groups] was tested using one-way ANOVA followed by post-hoc Dunnett's multiple comparisons test **(B–F)**, or Kruskal–Wallis followed by Dunn's multiple comparisons test **(A)**. *N* = 7–8 (sham *N* = 4). Results are shown as mean ± SEM (*p < 0.05, **p < 0.01).

Next, we measured Th2-related cytokine and chemokine levels in the lungs. In the BALF, concentrations of IL-5, IL-13, and CCL22 (Figures 3A–C), but not CCL5 or IFN-γ (Supplementary Figure S6), were reduced in at least one of the FOS intervention groups. However, only the 1% FOS diet significantly reduced IL-5 and CCL22, and the 1, 2.5, and 10% FOS diets reduced IL-13. The 5% and 10% FOS diets also tended (*p* = 0.095 and *p* = 0.050, respectively) to reduce CCL22 in BALF. In lung homogenates of HDM-allergic mice, several cytokine concentrations (IL-4, IL-5, IL-13, CCL5, CCL20, CCL22, IFN-γ, and IL-10) were increased compared to sham, but dietary FOS did not affect these levels (Supplementary Figure S7).

To study the allergen-specific T-cell response, lung cell suspensions were *ex vivo* restimulated with HDM. IL-13 was significantly increased in HDM-allergic mice compared to sham (Figure 3D), and IL-10 showed a similar tendency (*p* = 0.096) (Figure 3E). Compared to control diet-fed HDM-allergic mice, lung cells from mice that received the 10% FOS diet had significantly suppressed IL-13 production upon *ex vivo* HDM restimulation (Figure 3D). Concomitantly, these mice showed an increasing trend in IL-10 production, although it did not reach significance (Figure 3E). Yet, the IL-13/IL-10 ratio significantly reduced (Figure 3F). Lower doses of FOS showed a similar pattern, although significance was not reached. Cells exposed to medium did not show any cytokine release (Supplementary Figure S8), while IFNγ concentrations were below the detection limit. In summary, these results corroborate the phenotypic shift in the pulmonary T lymphocytes and show a transition in the cytokine profile, with a less Th2-dominant response.

### FOS affects the fecal microbiome of mice in association with protection against type 2 inflammation

**Figure 4:**
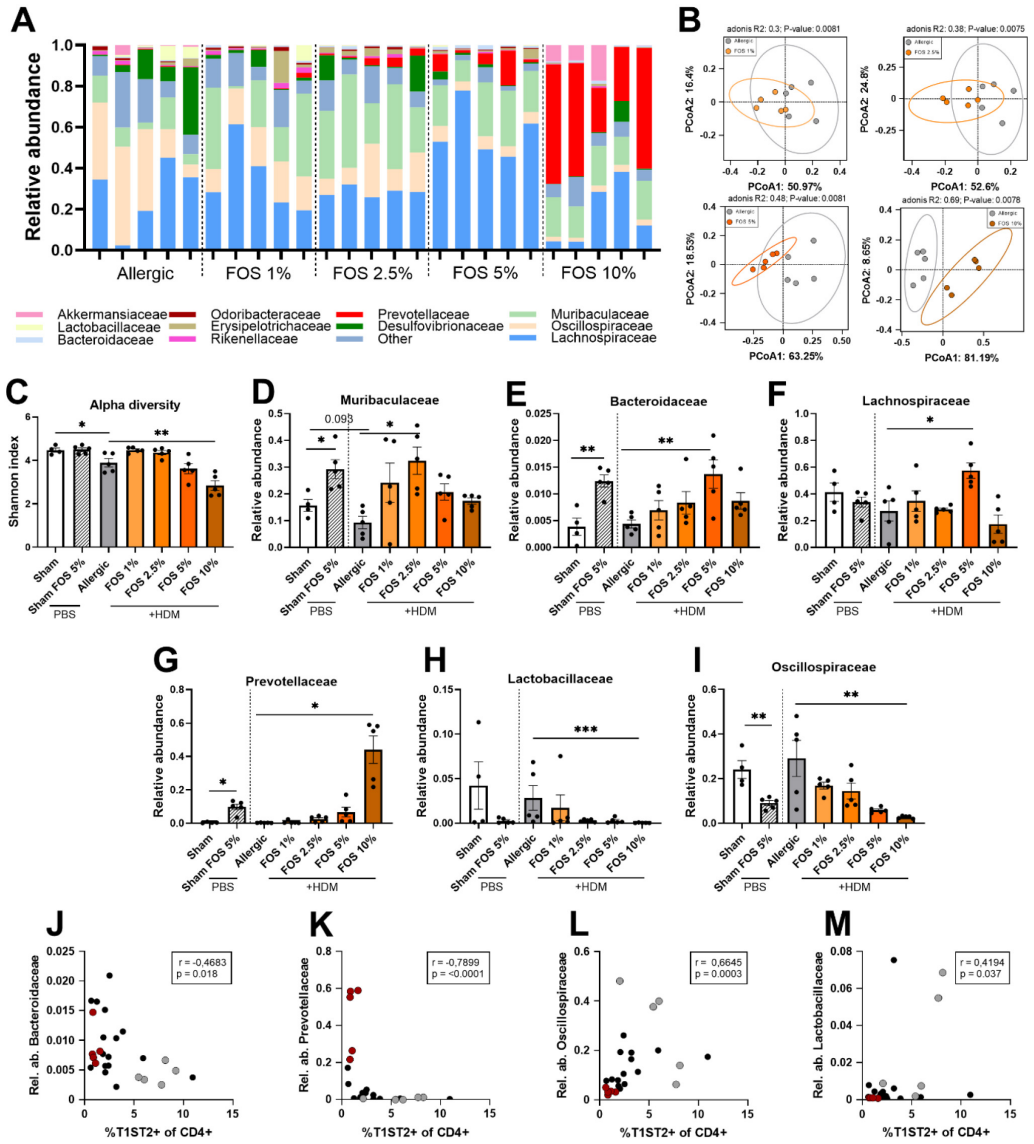
Effects of FOS on fecal microbiome composition in HDM-allergic mice and correlations of fecal microbiome with lung %Th2 cells. Fecal samples of a random selection of mice from each experimental group were analyzed using 16S rRNA gene-sequencing (sham *N* = 4, other *N* = 5). **(A)** Microbiome profiles of the HDM-allergic mice groups, depicted as relative abundance of the top 10 bacterial families found in the Fecal samples are displayed. **(B)** Principal Coordinates Analysis for beta diversity between the allergic group without FOS intervention and each dose of dietary FOS intervention, including Adonis value **(C)** Alpha diversity as determined by Shannon Index. Differences in the relative abundance of **(D)** Muribaculaceae, **(E)** Bacteroidaceae, **(F)** Lachnospiraceae, **(G)** Prevotellaceae, **(H)** Lactobacillaceae, and **(I)** Oscillospiraceae between the different intervention groups. Correlations of the frequency of Th2 cells (%T1ST2 of CD4+) with **(J)** Bacteroidaceae, **(K)** Prevotellaceae, **(L)** Lactobacillaceae, and **(M)** Oscillospiraceae. Gray dots are values of allergic (HDM) control diet-fed mice, red dots are values of HDM-exposed-10% FOS diet mice, and black dots are of HDM-exposed-1%/2.5%/5% FOS diet mice. For **(B)** beta diversity, a permutational multivariate analysis of variance (PERMANOVA) was performed. For C-I), the experimental model (aham vs. allergic), and sham vs. 5%F OS effect comparisons were analyzed using an unpaired t-test **(C, G)** experimental model, **(H)** or Mann–Whitney test [**(G)** Sham- Sham FOS5%]. The efect of the diets on HDM allergy [allergic (HDM)-control diet vs. allergic (HDM)-FOS diet groups] was tested using one-way ANOVA **(C, E, H)**, Brown–Forsythe and Welch ANOVA test **(D, F, G, I)** or Kruskal–Wallis test **(I)**, followed by post-hoc Dunnett's, Dunnett's T3 or Dunn's multiple comparisons test. Results are shown as mean ± SEM (*p < 0.05, **p < 0.01, ***p < 0.001). For **(J–M)**, Spearman r correlation matrices were obtained and significant correlations were visualized. *p* < 0.05 was considered significant. Data from all HDM-exposed mice (both the control diet and with FOS intervention) were included. *N* = 25.

Fecal microbial composition in allergic (Figure 4A) and sham (Supplementary Figure S9A) mice was determined using 16S rRNA-gene sequencing. Microbial community differences between samples were quantified based on weighted UniFrac distances. Adonis values (*R*^2^ statistic), reflecting the proportion of variation explained by the grouping variable, were calculated to compare the groups (beta-diversity). The higher the percentage of FOS in the diet was, the higher the Adonis value was found to be. Hence the proportion of the total variation in the data explained by dietary intervention with FOS, related to beta diversity in the fecal samples, increased with higher percentages of FOS in the diet (Figure 4B). All different FOS doses significantly explained the increase in Adonis values. Furthermore, the difference in beta diversity of the microbiome between sham mice and sham mice receiving 5% FOS was significant, as indicated by the Adonis value (Supplementary Figure S9B). Microbial diversity within the samples was assessed using the Shannon Index (combining both richness and evenness of the community into a single value). This microbial alpha diversity was significantly decreased in allergic mice compared to sham mice (Figure 4C). Dietary intervention with 10% FOS induced a further decrease in alpha diversity of the microbiome of HDM-allergic mice compared to HDM-allergic mice fed a control diet. The changes in microbial alpha and beta diversity in HDM-allergic mice receiving FOS can likely be explained by shifts in the presence, absence, and relative abundance of multiple bacterial families. While 1% FOS did not significantly alter the abundance of any specific bacterial family in HDM-allergic mice, 2.5% FOS significantly increased the abundance of *Muribaculaceae* (Figure 4D). A dietary intervention with 5% FOS significantly increased the abundance of *Bacteroidaceae* (Figure 4E) and *Lachnospiraceae* (Figure 4F). Finally, a 10% FOS diet significantly increased *Prevotellaceae* abundance (Figure 4G), while the abundance of *Lactobacillaceae* (Figure 4H) and *Oscillospiraceae* (Figure 4I) was significantly decreased. Dietary supplementation with 5% FOS in sham animals also altered the bacterial abundance, including increases in *Muribaculaceae, Bacteroidaceae*, and *Prevotellaceae* (Figures 4D, E, G) and a decrease in *Oscillospiraceae* (Figure 4I). Finally, it was observed that in various mice that received 10% FOS, *Akkermansiaceae* were present (Figure 4A), whereas this was not the case in mice receiving lower FOS doses. Interestingly, *Lachnospiraceae* abundance was not increased in sham mice fed 5% FOS, whereas this was increased in the HDM-allergic mice only in the 5% group (Figure 4F). Taken together, dietary supplementation with 2.5–10% FOS significantly altered the fecal microbiome composition. These microbial shifts were dose-dependent and associated with specific changes in bacterial family abundance, including increases in *Muribaculaceae, Bacteroidaceae, Lachnospiraceae*, and *Prevotellaceae*, decreases in *Lactobacillaceae* and *Oscillospiraceae*, and the appearance of *Akkermansiaceae* only at the 10% FOS dose.

As we observed dose-dependent shifts both in the type 2 inflammation profile and in the fecal microbial composition, we examined correlations between microbiome composition and SCFAs with markers of type 2 inflammation in HDM-exposed mice (0, 1, 2.5, 5, and 10% FOS, all combined). A low frequency of Th2 cells in the lungs correlated with increased abundance of *Bacteroidaceae* (Figure 4J) and *Prevotellaceae* (Figure 4K), and decreased abundance of *Oscillospiraceae* (Figure 4L) and *Lactobacillaceae* (Figure 4M). Table 2 shows additional correlations between type 2 immune-parameters and microbial composition. In HDM-allergic mice fed higher doses of FOS, *Bacteroidaceae* tended to correlate positively with IL-10 (*p* = 0.097), while *Lactobacillaceae* and *Oscillospiraceae* correlated negatively. *Prevotellaceae* was negatively correlated with the percentage of lymphocytes in the BALF, the Th2/Th1 ratio in the lung, and IL-13 release upon *ex vivo* HDM restimulation of lung cells (Table 2). By contrast, *Lachnospiraceae* were positively correlated with the ratio of activated Th2/Th1 cells in the lung, and *Lactobacillaceae, Oscillospiraceae*, and *Lachnospiraceae* showed a similar tendency with the overall Th2/Th1 ratio in the lung (*p* = 0.067, *p* = 0.023, and *p* = 0.089, respectively) (Table 2). Thus, *Bacteroidaceae* and *Prevotellaceae* showed a protective correlation with type 2 allergic immune outcomes, whereas *Lactobacillaceae, Oscillospiraceae*, and *Lachnospiraceae* appeared more unfavorable. *Prevotellaceae* showed the strongest type 2 protective effect, as it correlated with multiple type 2 markers (Table 2). Altogether, these correlations show that, particularly *Bacteroidaceae* and *Prevotellaceae*, which increased in abundance after dietary intervention with 5% and 10% FOS, respectively, are strongly and inversely correlated with markers of type 2 inflammation in HDM-allergic mice. In contrast, *Lachnospiraceae*, which was stimulated by 5% FOS, correlated with increased type 2 inflammatory markers.

**Table 2:** Correlations (Spearman *r*) between immune-parameters and microbiome (fermentation) parameters^a^.

**Microbiome (fermentation) parameter^e^**	**Immune-parameter**							
	**Lung**^b^ **%T1ST2**+ **of CD4**+	**BALF**^c^ **%Total lymphocytes**	**Lung**^b^ **ratio Th2/Th1**	**Lung**^b^ **ratio activated Th2/Th1**	**BALF**^c^ **IL-13 (pg/mL)**	**Restim**^d^ **IL-13 (pg/mL)**	**Restim**^d^ **IL-10 (pg/mL)**	**Restim**^d^ **IL-13/IL-10**
*Bacteroidaceae*	−0.468^*^	–	–	–	–	–	0.339^#^	–
*Prevotellaceae*	−0.790^*^	−0.437^*^	−0.515^**^	–	–	−0.42^*^		−0.499^*^
*Lactobacillaceae*	0.419^*^	–	0.372^#^	–	–	–	−0.542^**^	–
*Oscillospiraceae*	0.665^***^	–	0.453^*^	–	–	–	−0.484^*^	–
*Lachnospiraceae*	–	–	0.348^#^	0.449^*^	–	–	–	–
*Muribaculaceae*	–	–	–	–	–	–	–	–
*Cecum acetate*	−0.451^**^	–	–	–	–	–	–	–
*Cecum propionate*	−0.373^*^	–	–	–	–	–	–	–
*Cecum butyrate*	−0.482^**^	–	–	–	–	–	–	–
*Serum acetate*	–	–	−0.367^*^	−0.375^*^	−0.347^*^	-	-	−0.343^*^
*Serum propionate*	–	–	–	–	–	–	–	–
*Serum butyrate*	−0.301^#^	–	–	–	–	–	–	–

^a*^p < 0.05, ^**^p < 0.01, ^***^p < 0.001. ^#^*p* < 0.1.

^b^From lung cell suspension.

^c^Bronchoalveolar lavage fluid. Total lymphocytes determined by flow cytometry.

^d^Lung cell suspension *ex vivo* restimulated with HDM, concentrations measured in supernatant.

^e^Bacterial families as relative abundance in the feces, Cecum SCFAs = μmol/g cecum content. Serum SCFAs = μM.

### FOS dose-dependently increases cecal SCFAs and serum acetate concentrations link with type 2 protection in the lungs

**Figure 5:**
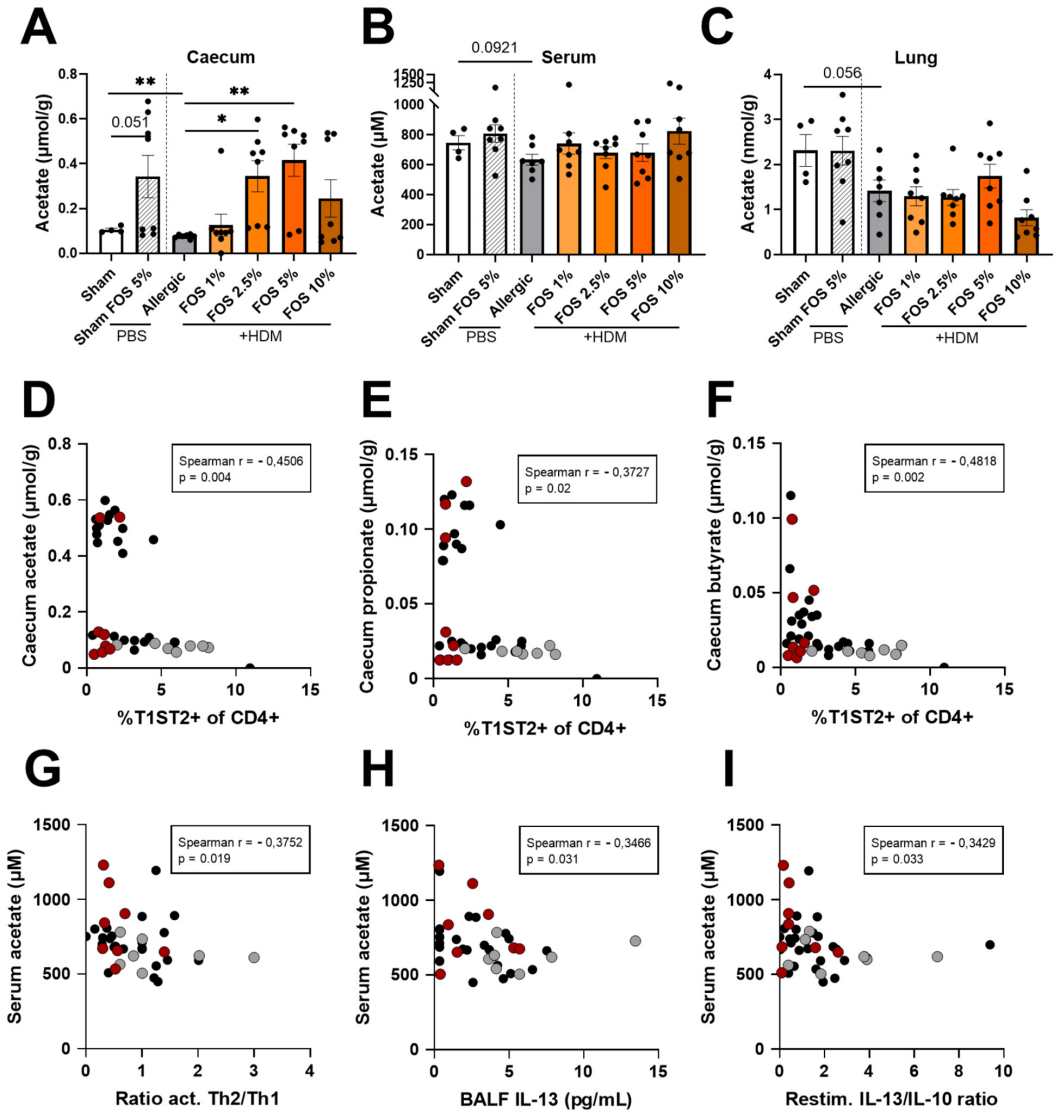
Acetate levels in cecum content, serum, and lung, and correlations between SCFA-levels and immune-parameters. Acetate levels were quantified in **(A)** cecum content, **(B)** serum, and **(C)** lung, using liquid chromatography-tandem mass spectrometry (LC-MS/MS). Correlations of the frequency of Th2 cells (%T1ST2 of CD4+) with **(D)** Cecum acetate, **(E)** Cecum propionate, and **(F)** Cecum butyrate. Correlation of acetate with **(G)** the ratio activated Th2/Th1 in the lung, **(H)** IL-13 produced by lung cells upon ex vivo HDM restimulation, and **(I)** the ratio IL-13/IL-10 produced by lung cells upon ex vivo HDM restimulation. In panels **(D–I)**, gray dots are values HDM-exposed-0% FOS diet mice, red dots are values HDM-exposed-10% FOS diet mice, and black dots are HDM-exposed-1%/2.5%/5% FOS diet mice. For **(A–C)**, the experimental model (sham vs. sllergic), and sham vs. 5% FOS effect comparisons were analyzed using an unpaired t-test [**(A)** experimental model, **(B)** and **(C)**] or Welch's t-test [**(A)** sham-sham FOS 5%]. The effect of the diets on HDM allergy [allergic (HDM)-control diet vs. Allergic (HDM)-FOS diet groups] was tested using one-way ANOVA [**(B)** and **(C)** or Kruskal–Wallis test **(A)**], followed by post-hoc Dunnett's or Dunn's multiple comparisons test. *N* = 4–8. Results are shown as mean ± SEM (*p < 0.05, **p < 0.01). For **(D–I)**, Spearman r correlation matrices were obtained and significant correlations were visualized. *N* = 39. *p* < 0.05 was considered significant. Data from all HDM-exposed mice (both control diet and with FOS intervention) were included.

In addition to changes in the fecal microbial composition, intestinal (cecum content), systemic (serum), and lung SCFA levels were determined. Cecal acetate levels were significantly decreased in control-fed HDM-exposed mice compared to sham (Figure 5A). After receiving the 2.5% and 5% FOS diets (both in sham and allergic (HDM) mice), cecal acetate levels were significantly increased. Although in control diet-fed allergic mice serum acetate levels again showed a decreasing trend (*p* = 0.092) compared to sham, none of the FOS diets affected serum acetate levels (Figure 5B). A similar pattern was observed for lung acetate levels (Figure 5C). Propionate levels in cecum content, serum, and lung followed the same pattern as observed for acetate (Supplementary Figures S10A–C). Butyrate levels in cecum content were decreased in control diet-fed allergic mice compared to sham (Supplementary Figure S10D), while only the 5% FOS diet significantly increased cecal butyrate levels. In the serum, butyrate levels were increased in mice fed 5% FOS (Supplementary Figure S10E). Lung butyrate levels were not increased by the 5% FOS diet in HDM-exposed mice, but were increased in sham mice fed the 5% FOS diet (Supplementary Figure S10F). Overall, the 5% FOS diet was most effective in increasing SCFA levels, particularly in cecum content and serum.

Beyond specific bacterial families, fermentation products were also be correlated to type 2 allergic asthma outcomes in HDM-exposed mice. Cecal acetate (Figure 5D), propionate (Figure 5E), and butyrate (Figure 5F) levels correlated negatively with Th2 cell frequency in the lungs. Furthermore, serum acetate concentrations were correlated negatively with the ratio of Th2/Th1 cells (*r* = −0.3752, *p* = 0.019; Spearman), the ratio of activated Th2/Th1 cells (Figure 5G) in the lung, IL-13 concentrations in the BALF (Figure 5H), and the IL-13/IL-10 ratio upon *ex vivo* HDM restimulation of lung cells (Figure 5I). All the above correlations suggest a protective role for increasing serum acetate levels against type 2 inflammation in the lung. Serum butyrate tended to negatively correlate with the Th2/Th1 ratio in the lungs (*p* = 0.063) (Table 2).

Taking all observations into account, supplying HDM-exposed mice with FOS, especially doses of 5% and 10%, affected the intestinal microbial composition and SCFA levels in several compartments. The most prominent changes induced by these diets, including the increased prevalence of *Prevotellaceae* and elevated levels of serum acetate, were correlated with the type 2 immune-suppressing effects of the same diets, emphasizing the role of the gut-lung axis.

To link bacterial families to metabolite (SCFA) profiles, we examined potential correlations (Table 3). Serum acetate concentrations were positively correlated only with *Prevotellaceae* abundance (Table 3). *Prevotellaceae* also correlated positively with serum propionate and negatively with lung acetate levels. By contrast, *Lachnospiraceae* correlated negatively with serum acetate and propionate and positively with lung acetate levels (Table 3).

**Table 3:** Correlations (Spearman *r*) between bacterial families in the *feces* (relative abundance) and short-chain fatty acid levels in different compartments^a^.

**Relative abundance bacterial family^c^**	**Short-chain fatty acid levels** ^**b**^								
	**Cecum cetate**	**Cecum propionate**	**Cecum butyrate**	**Serum acetate**	**Serum propionate**	**Serum butyrate**	**Lung acetate**	**Lung propionate**	**Lung butyrate**
*Prevotellaceae*	–	–	–	0.590^**^	0.444^*^	-	−0.441^*^	–	–
*Bacteroidaceae*	–	–	0.425^*^	–	–	–	–	–	–
*Muribaculaceae*	0.593^**^	0.489^*^	0.545^**^	–	–	–	–	–	–
*Lachnospiraceae*	–	–	-	−0.435^*^	−0.390^#^	-	0.419^*^	–	–
*Oscillospiraceae*	–	–	−0.348^#^	–	–	–	–	–	–
*Lactobacillaceae*	–	–	–	–	–	–	–	–	–

^a*^p < 0.05, ^**^p < 0.01, ^#^*p* < 0.1.

^b^ Cecum SCFAs = μmol/g cecum content. Serum SCFAs = μM. Lung SCFAs = nmol/g lung.

^c^Bacterial families as relative abundance in the faeces.

*Bacteroidaceae* correlated positively only with cecal butyrate, while *Oscillospiraceae* tended to negatively correlate with cecal butyrate (*p* = 0.089). *Muribaculaceae* positively correlated to cecal acetate, propionate, and butyrate (Table 3).

## Discussion

Dietary fibers, such as fructo-oligosaccharides (FOS), play a crucial role in gut microbiome and immune homeostasis and influence the pathophysiology of allergic asthma (24). The heterogeneity of the effects of specific dietary fibers (24) or specific dosing (15), along with the disease-context-dependent definition of a “healthy” microbiome (9), highlights the need for well-defined experiments to study the preventative effects of dietary fibers on allergic asthma. We investigated the effects of dietary supplementation with four different doses (1, 2.5, 5, and 10%) of FOS (1:1 short-chain: long-chain ratio) on the development of acute HDM-induced allergic airway inflammation and on the microbiome composition and function in mice.

Although previous studies using a combination of 1% (w/w) FOS (1:1) with *Bifidobacterium* breve or 1% (w/w) galacto-oligosaccharides (GOS) largely prevented both eosinophilic airway inflammation and type 2 inflammation in an acute HDM-induced murine asthma model (16–18), in the current study, 1% (w/w) FOS did not protect against type 2 inflammation. A higher dose of FOS (10%) did dampen the Th2 effector response, as shown by a shift in the Th2/Th1 balance and reduced IL-13 production, although markers of allergic sensitization (HDM-IgE) and eosinophilic airway inflammation were not affected by this diet. The dissociation between these findings may reflect a subtle immunomodulatory effect of FOS supplementation, with the intervention predominantly acting on localized modulation of T-cell responses in the lung, without exerting widespread control over the influx or activation of other immune cells driving allergic inflammation. The mechanisms underlying this apparent dissociation between tissue-level and airway inflammatory changes remain unclear, but possible explanations include specific metabolites or signaling molecules in the gut-lung axis that may primarily affect T-cell differentiation and/or function, possibly via modulation of dendritic cell function. Previous studies have shown bacterial metabolites to modulate dendritic cell development in the bone marrow, yielding a less inflammatory phenotype with reduced capacity to drive HDM-induced Th2 inflammation (19).

Other studies using different dietary fibers, such as pectin and high-amylose starch at substantially higher doses (30% and 72.3% w/w, respectively), reported either inhibition of airway eosinophilia without changes in lymphocyte frequency (19), or a decrease in both cell types (21). These discrepancies highlight the fiber-specific and dose-dependent nature of gut-lung axis modulation.

Alterations in the intestinal microbiome were associated with immunomodulatory effects in the lung. In particular, the 10% FOS diet increased *Prevotellaceae* abundance while decreasing *Oscillospiraceae* and *Lactobacillaceae* abundance, all of which correlated with protective effects on Th2 development. Although these parameters were related, these correlations do not necessarily entail causation. Nevertheless, they can act as sources for future hypothesis-driven research, in which the effects of enrichment with, or exclusion of, these bacteria on the immune system could be further elucidated. *Prevotellaceae* are well-known fiber fermenters (including inulin) (25–27) and associated with a plant-rich, non-Western diet (28, 29). The genus *Prevotella* (*or Segatella*), particularly *Prevotella copri*, is reduced in individuals with asthma (30) and allergic rhinitis (31). Increased abundance is associated with protection against several allergic diseases (32–34), including asthma (35). However, depending on the subtype of *Prevotellaceae*, this genus has also been found to positively associate with childhood asthma (36). Microbial analyses at a lower taxonomic level and intervention studies with *Prevotellaceae* subspecies supplementation could further deepen our understanding of its possible role in beneficial gut–lung interactions, as suggested by our data in young-adult mice. Notably, the 10% FOS diet also increased the presence of *Akkermansia* bacteria in some mice. *Akkermansia muciniphila* is generally related to positive health outcomes, including a suggested protective role in allergic asthma based on mouse studies (37). This observation aligns with our findings of some immunomodulatory effects of the 10% FOS diet.

In contrast to *Prevotellaceae, Oscillospiraceae* and *Lactobacillaceae* were associated with increased type 2 inflammation. Although typically reduced in type 2 asthma (38, 39), their abundance decreased only in HDM-exposed mice fed the 10% FOS diet, and not when fed the control diet. This suggests that *Prevotellaceae* may have outcompeted these taxa and exerted stronger positive effects. FOS is generally reported to be a bifidogenic fiber in humans (40). Although *Bifidobacteriaceae* were detected in the current study, no bifidogenic effect was observed. This is in line with previous murine studies and is likely due to a low initial abundance and the age of the mice (41–43).

Lower FOS doses (2.5 and 5%) also modified the gut microbiota, although these changes did not confer protection against type 2 airway inflammation, as observed with 10% FOS. The 2.5% FOS diet increased *Muribaculaceae*, which correlated with higher cecal SCFA levels. Despite the negative association between SCFAs and lung Th2 cell frequencies, this effect was not strong enough to reduce type 2 inflammation. *Muribaculaceae* are prominent fiber metabolizers in mice and produce SCFAs that cross-feed *Bifidobacterium* and *Lactobacillus*, but their role in disease regulation remains unclear (44).

In the 5% FOS group, *Bacteroidaceae* abundance and cecal and serum butyrate levels increased, suggesting that this family may contribute to FOS fermentation into SCFAs. Notably, in humans, the main genus within the *Bacteroidaceae* family, *Bacteroides*, is reported to decrease after inulin supplementation (45), and in general, the genus tends to be inversely related to *Prevotella* (46, 47). At the same, *Lachnospiraceae* were enriched and correlated positively with the Th2/Th1 ratio and negatively with serum acetate and propionate, potentially offsetting beneficial microbial effects. Notably, *Lachnospiraceae* have been positively associated with inflammatory conditions such as metabolic syndrome and inflammatory bowel disease (48) and allergic infants (49), but negatively associated with asthma in adults (38).

Typically, 5% FOS enhanced *Muribaculaceae, Bacteroidaceae*, and *Prevotellaceae* in sham mice, whereas in HDM-exposed mice, 5% FOS increased *Bacteroidaceae* and *Lachnospiraceae*. Thus, HDM exposure itself also interfered with the microbiome modulation induced by 5% FOS. Although underlying mechanisms remain unclear, it has been hypothesized that HDM allergens reaching the gut may compromise the integrity of the gut epithelial barrier, triggering gut dysfunction (50) and potentially affecting the microbiome. Consequently, it is plausible that the microbiota-modulating effects of FOS differ between control and HDM-exposed conditions.

FOS-induced SCFA levels, especially in the cecum content, followed an inverted U-shape pattern, with the highest levels detected at a dose of 5% FOS. A study in rats, in which 0, 5, 10, and 20% inulin (w/w) were provided, also revealed an inverted U-shape pattern regarding acetate, propionate, and butyrate concentrations in the feces, with the optimum at 10% inulin (15). In the current study, increased concentrations of serum acetate and propionate were associated with less type 2 inflammation. Previously, Trompette et al. (19) demonstrated that propionate in drinking water protects mice from HDM-induced type 2 allergic inflammation. Compared to propionate and butyrate, acetate has been less studied in the context of immune modulation. Nevertheless, acetate is recognized for its biological activity through binding to G-protein-coupled receptor 43 (GPR43), inhibiting histone deacetylases (HDACs), and suppressing NF-κβ activation. Together, these mechanisms can influence immune function (51), which was shown for example in mouse models of allergic asthma (19, 21).

Interestingly, although the 5% FOS diet showed more pronounced SCFA production, it had less protective impact on type 2 inflammation than the 10% FOS diet. Our data suggest that the SCFAs acetate, propionate, and butyrate are not the only responsible players when studying the effects of FOS fermentation by the gut microbiome on HDM-induced type 2 allergic inflammation in mice. Furthermore, other microbial metabolites, such as indole-3-propionic acid (52), p-cresol (53), and its sulfation product p-cresol sulfate (54), are associated with a protective effect on allergic asthma development. For example, antibiotics induced dysbiosis results in loss of indole-3-propionic acid production, while supplying this via drinking water could protect mice from severe asthma development by preventing mitochondrial stress in bronchial epithelial cells upon HDM exposure (52). However, as these specific examples are derivatives of amino acids, tryptophan or tyrosine, FOS cannot directly be linked to these fermentation products. Nevertheless, FOS may enhance colonization of bacterial species linked to the metabolism of these essential amino acids, such as the unclassified *Prevotella MGM1* (52). However, this species was not assessed in our microbiome analyses. Other microbiome metabolites known to modify type 2 inflammation in preclinical models include, among others, secondary bile acids, sphingolipids, and histamine (55, 56). Notably, the current study is not the first describing that different doses and degrees of polymerization of FOS have different microbial preferences and microbiome changes (47, 57, 58), and therefore metabolic formation and immune modulation (57). Furthermore, increasing FOS doses may have affected FOS fermentation, yielding smaller FOS molecules available for cross-feeding (59). Since this field is evolving rapidly, mechanistic cause-and-effect studies are warranted to fully appreciate their potency in asthma risk or protection. FOS suppressed cytokine responses in BALF and reduced allergen-specific restimulation of lung cell suspensions, while cytokine expression of lung homogenates remained unaltered. This implies selective modulation of certain immune cell types, including T-cells, while leaving other type 2 cytokine-producing tissue-resident cells, such as ILCs and mast cells, unaffected. Indeed, the allergen-specific cytokine responses mainly represent T-cell responses upon activation by allergen-presenting cells. T-cells may be more sensitive to the effects of SCFA as these may act via GPR41/43 receptors on T-cells, while mast cell and ILC function is mainly modified via HDAC inhibition, which may require higher SCFA doses (12).

Several limitations should be considered when interpreting our findings. Although our results suggest potential links between FOS-modulated microbes (e.g., *Prevotellaceae*), acetate levels, and Th2 responses, functional validation—such as supplementation with specific SCFAs, microbiota depletion, or microbial transfer into germ-free or antibiotic-treated mice—will be required to confirm causality in these interactions and elucidate the underlying mechanisms.

This study aimed to identify if there is an optimal FOS dose for asthma prevention; only male mice were used. However, asthma pathophysiology (60) and gut microbiota composition (61) are known to be influenced by sex hormones, and future studies including both sexes will be important to fully understand FOS effects on the gut–lung axis.

While 16S rRNA sequencing provided valuable taxonomic insight, functional prediction or pathway-based analyses would help clarify the metabolic consequences of FOS-induced microbial shifts and strengthen mechanistic interpretation. Moreover, the translation of murine microbiome data to humans remains challenging due to interspecies and inter-strain differences in microbial composition (62). Functional profiling approaches focusing on metabolic pathways and biological processes may enhance translatability (63).

Finally, this study primarily assessed immunological parameters. Incorporating functional and histopathological measurements in future studies will further clarify how these immune alterations relate to airway physiology and tissue remodeling.

This study reveals that the immunomodulatory effects of FOS on allergic airway inflammation are not solely dependent on SCFA abundance, but rather on a complex FOS dose-dependent interplay between microbial composition, metabolite distribution, and systemic immune regulation. The protective effect observed with the 10% FOS diet indicates that specific microbial shifts—particularly the enrichment of *Prevotellaceae*—may be linked to partial protection from type 2 inflammation, potentially through serum acetate-mediated pathways. These findings challenge the conventional focus on gut SCFA levels as primary mediators and instead highlight the importance of microbial ecology and systemic metabolite availability, and their modulation in shaping immune outcomes. Ultimately, our data support the concept that targeted dietary interventions can recalibrate the gut–lung axis, offering a promising avenue for the prevention or mitigation of allergic asthma.

## Data Availability

The data presented in this study are publicly available. The data can be found here: https://www.ncbi.nlm.nih.gov/sra, accession PRJNA1433716.

## References

[1] The Global Asthma Network. The global asthma report 2022. Int J Tubercul Lung Dis. (2022) 26:S1–102. doi: 10.5588/ijtld.22.1010

[2] Hammad H Lambrecht BN. The basic immunology of asthma. Cell Elsevier BV. (2021) 184:1469–85. doi: 10.1016/j.cell.2021.02.016 33711259

[3] Haahtela T Jantunen J Saarinen K Tommila E Valovirta E Vasankari T . Managing the allergy and asthma epidemic in 2020s—lessons from the Finnish experience. Allergy. (2022) 77:2367–80. doi: 10.1111/all.15266 35202479 PMC9546028

[4] Haahtela T Holgate S Pawankar R Akdis CA Benjaponpitak S Caraballo L . The biodiversity hypothesis and allergic disease: world allergy organization position statement. World Allergy Organiz J. (2013) 6:3. doi: 10.1186/1939-4551-6-3 23663440 PMC3646540

[5] Ogulur I Mitamura Y Yazici D Pat Y Ardicli S Li M . Type 2 immunity in allergic diseases. Cell Mol Immunol. (2025) 22:211. doi: 10.1038/s41423-025-01261-2 39962262 PMC11868591

[6] Frati F Salvatori C Incorvaia C Bellucci A Di Cara G Marcucci F . The role of the microbiome in asthma: the gut–lung axis. Int J Mol Sci. (2019) 20:1–12. doi: 10.3390/ijms20010123 30598019 PMC6337651

[7] Seluk L Davis AE Rhoads S Wechsler ME. Novel asthma treatments: Advancing beyond approved novel step-up therapies for asthma. Ann Allergy Asthma Immunol. (2025) 134:9–18. doi: 10.1016/j.anai.2024.09.016 39393433

[8] Papadopoulos NG Miligkos M Xepapadaki P. A current perspective of allergic asthma: from mechanisms to management. Handb Exp Pharmacol. (2022) 268:69–93. doi: 10.1007/164_2021_483 34085124

[9] Joos R Boucher K Lavelle A Arumugam M Blaser MJ Claesson MJ . Examining the healthy human microbiome concept. Nat Rev Microbiol. (2024) 23:192–205. doi: 10.1038/s41579-024-01107-0 39443812

[10] Haahtela T Laatikainen T Alenius H Auvinen P Fyhrquist N Hanski I . Hunt for the origin of allergy - comparing the Finnish and Russian Karelia. Clin Exp Allergy. (2015) 45:891–901. doi: 10.1111/cea.12527 25772429

[11] Ross FC Patangia D Grimaud G Lavelle A Dempsey EM Ross RP . The interplay between diet and the gut microbiome: implications for health and disease. Nat Rev Microbiol. (2024) 22:671–86. doi: 10.1038/s41579-024-01068-4 39009882

[12] Mann ER Lam YK Uhlig HH. Short-chain fatty acids: linking diet, the microbiome and immunity. Nat Rev Immunol. (2024) 24:577–95. doi: 10.1038/s41577-024-01014-8 38565643

[13] Marinelli L Martin-Gallausiaux C Bourhis JM Béguet-Crespel F Blottière HM Lapaque N. Identification of the novel role of butyrate as AhR ligand in human intestinal epithelial cells. Sci Rep. (2019) 9:643. doi: 10.1038/s41598-018-37019-2 30679727 PMC6345974

[14] Tan JK Macia L Mackay CR. Dietary fiber and SCFAs in the regulation of mucosal immunity. J Allergy Clin Immunol. (2023) 151:361–70. doi: 10.1016/j.jaci.2022.11.007 36543697

[15] Levrat MA. High propionic acid fermentations and mineral accumulation in the cecum of rats adapted to different levels of inulin. J Nutr. (1991) 121:1730–7. 1941180 10.1093/jn/121.11.1730

[16] Verheijden KA Willemsen LE Braber S Leusink-Muis T Delsing DJ Garssen J . Dietary galacto-oligosaccharides prevent airway eosinophilia and hyperresponsiveness in a murine house dust mite-induced asthma model. Respir Res. (2015) 16:17. doi: 10.1186/s12931-015-0171-0 25849971 PMC4327967

[17] Verheijden KA Willemsen LE Braber S Leusink-Muis T Jeurink PV Garssen J . The development of allergic inflammation in a murine house dust mite asthma model is suppressed by synbiotic mixtures of non-digestible oligosaccharides and Bifidobacterium breve M-16V. Eur J Nutr. (2016) 55:1141–51. doi: 10.1007/s00394-015-0928-8 26003185 PMC4819948

[18] Verheijden KAT Braber S Leusink-Muis T Jeurink PV Thijssen S Kraneveld AD . The combination therapy of dietary galacto-oligosaccharides with budesonide reduces pulmonary Th2 driving mediators and mast cell degranulation in a murine model of house dust mite induced asthma. Front Immunol. (2018) 9:2419. doi: 10.3389/fimmu.2018.02419 30405619 PMC6207001

[19] Trompette A Gollwitzer ES Yadava K Sichelstiel AK Sprenger N Ngom-Bru C . Gut microbiota metabolism of dietary fiber influences allergic airway disease and hematopoiesis. Nat Med. (2014) 20:159–66. doi: 10.1038/nm.3444 24390308

[20] Lewis G Wang B Shafiei Jahani P Hurrell BP Banie H Aleman Muench GR . Dietary fiber-induced microbial short chain fatty acids suppress ILC2-dependent airway inflammation. Front Immunol. (2019) 10:2051. doi: 10.3389/fimmu.2019.02051 31620118 PMC6760365

[21] Thorburn AN McKenzie CI Shen S Stanley D Macia L Mason LJ . Evidence that asthma is a developmental origin disease influenced by maternal diet and bacterial metabolites. Nat Commun. (2015) 6:1–13. doi: 10.1038/ncomms8320 26102221

[22] Verstegen REM Sparidans RW Kostadinova AI Garssen J Folkerts G Hendriks RW . Lung acetate levels decline in correlation with increased type 2 allergic markers in a house dust mite allergic mouse model. Clin Transl Allergy. (2025) 15:e70082. doi: 10.1002/clt2.70082 40760338 PMC12321596

[23] Zuurveld M de Kleer JWM Berends AJ Kooy MM Van Ark I Leusink-Muis T . HMOS 2'FL and 3FL prevent house dust mite induced proinflammatory cytokine release *in vitro* and decrease specific IgE production in a murine allergic asthma model. Front Nutr. (2025) 12:1491430. doi: 10.3389/fnut.2025.1491430 40046758 PMC11879794

[24] Verstegen REM Kostadinova AI Merenciana Z Garssen J Folkerts G Hendriks RW . Dietary fibers: effects, underlying mechanisms and possible role in allergic asthma management. Nutrients. (2021) 13:1–32. doi: 10.3390/nu13114153 34836408 PMC8621630

[25] Chen J Li Z Wang X Fan B Deng F D Yu H . Isomaltooligosaccharides sustain the growth of prevotella both *in vitro* and in animal models. Microbiol Spectr. (2022) 10:e02621–21. doi: 10.1128/spectrum.02621-21 36377936 PMC9769830

[26] Chen T Long W Zhang C Liu S Zhao L Hamaker BR. Fiber-utilizing capacity varies in *Prevotella*- versus *Bacteroides*-dominated gut microbiota. Sci Rep. (2017) 7:1–7. doi: 10.1038/s41598-017-02995-4 28572676 PMC5453967

[27] Kaoutari A El Armougom F Gordon JI Raoult D Henrissat B. The abundance and variety of carbohydrate-active enzymes in the human gut microbiota. Nat Rev Microbiol. (2013) 11:497–504. doi: 10.1038/nrmicro3050 23748339

[28] Yeoh YK Sun Y Ip LYT Wang L Chan FKL Miao Y . Prevotella species in the human gut is primarily comprised of *Prevotella copri, Prevotella stercorea* and related lineages. Sci Rep. (2022) 12:9055. doi: 10.1038/s41598-022-12721-4 35641510 PMC9156738

[29] Tett A Huang KD Asnicar F Fehlner-Peach H Pasolli E Karcher N . The *Prevotella copri* complex comprises four distinct clades underrepresented in westernized populations. Cell Host Microbe. (2019) 26:666–79.e7. doi: 10.1016/j.chom.2019.08.018 31607556 PMC6854460

[30] Mahdavinia M Fyolek JP Jiang J Thivalapill N Bilaver LA Warren C . Gut microbiome is associated with asthma and race in children with food allergy. J Allergy Clin Immunol. (2023) 152:1541. doi: 10.1016/j.jaci.2023.07.024 37714436 PMC10872992

[31] Sahoyama Y Hamazato F Shiozawa M Nakagawa T Suda W Ogata Y . Multiple nutritional and gut microbial factors associated with allergic rhinitis: the Hitachi Health Study. Sci Rep. (2022) 12:3359. doi: 10.1038/s41598-022-07398-8 35233003 PMC8888718

[32] Jungles K Tran TDB Botha M Rasmussen HE Teixeira-Reis V Sodergren E . Association of gut microbiota and environment in children with AD, comparison of three cohorts of children. Clin Exp Allergy. (2022) 52:447–50. doi: 10.1111/cea.14052 34786779

[33] Goldberg MR Mor H Magid Neriya D Magzal F Muller E Appel MY . Microbial signature in IgE-mediated food allergies. Genome Med. (2020) 12:92. doi: 10.1186/s13073-020-00789-4 33109272 PMC7592384

[34] Vuillermin PJ O'Hely M Collier F Allen KJ Tang MLK Harrison LC . Maternal carriage of *Prevotella* during pregnancy associates with protection against food allergy in the offspring. Nat Commun. (2020) 11:1452. doi: 10.1038/s41467-020-14552-1 32210229 PMC7093478

[35] Liu A Ma T Xu N Jin H Zhao F Kwok LY . Adjunctive probiotics alleviates asthmatic symptoms via modulating the gut microbiome and serum metabolome. Microbiol Spectr. (2021) 9:e00859–21. doi: 10.1128/Spectrum.00859-21 34612663 PMC8510161

[36] Yan T Bao Y Cao S Jiang P Zhang Z Li L . The investigation of the role of oral-originated *Prevotella*-induced inflammation in childhood asthma. Front Microbiol. (2024) 15:1400079. doi: 10.3389/fmicb.2024.1400079 38863747 PMC11165567

[37] Panzetta ME Valdivia RH. Akkermansia in the gastrointestinal tract as a modifier of human health. Gut Microbes. (2024) 16:2406379. doi: 10.1080/19490976.2024.2406379 39305271 PMC11418289

[38] Gu BH Choi JP Park T Kim AS Jung HY Choi DY . Adult asthma with symptomatic eosinophilic inflammation is accompanied by alteration in gut microbiome. Allergy: Eur J Allergy Clin Immunol. (2023) 78:1909–21. doi: 10.1111/all.15691 36847620

[39] Zimmermann P Messina N Mohn WW Finlay BB Curtis N. Association between the intestinal microbiota and allergic sensitization, eczema, and asthma: a systematic review. J Allergy Clin Immunol. (2019) 143:467–85. doi: 10.1016/j.jaci.2018.09.025 30600099

[40] Dou Y Yu X Luo Y Chen B Ma D Zhu J. Effect of fructooligosaccharides supplementation on the gut microbiota in human: a systematic review and meta-analysis. Nutrients. (2022) 14:3298. doi: 10.3390/nu14163298 36014803 PMC9413759

[41] Muthyala SDV Shankar S Klemashevich C Blazier JC Hillhouse A Wu CS. Differential effects of the soluble fiber inulin in reducing adiposity and altering gut microbiome in aging mice. J Nutr Biochem. (2022) 105:108999. doi: 10.1016/j.jnutbio.2022.108999 35346831

[42] An R Zhou X Zhang J Lyu C Wang D. Responses of intestinal microbiota to inulin was initial microbiota context dependent and affected by the supplementation dosage. Food Res Int. (2025) 200:115498. doi: 10.1016/j.foodres.2024.115498 39779139

[43] Zhu Z Hu C Liu Y Wang F Zhu B. Inulin has a beneficial effect by modulating the intestinal microbiome in a BALB/c mouse model. Benef Microbes. (2023) 14:371–83. doi: 10.1163/18762891-20220094 38661353

[44] Zhu Y Chen B Zhang X Akbar MT Wu T Zhang Y . Exploration of the muribaculaceae family in the gut microbiota: diversity, metabolism, and function. Nutrients. (2024) 16:2660. doi: 10.3390/nu16162660 39203797 PMC11356848

[45] Le Bastard Q Chapelet G Javaudin F Lepelletier D Batard E Montassier E. The effects of inulin on gut microbial composition: a systematic review of evidence from human studies. Eur J Clin Microbiol Infect Dis. (2020) 39:403–13. doi: 10.1007/s10096-019-03721-w 31707507

[46] Gorvitovskaia A Holmes SP Huse SM. Interpreting Prevotella and Bacteroides as biomarkers of diet and lifestyle. Microbiome. (2016) 4:15. doi: 10.1186/s40168-016-0160-7 27068581 PMC4828855

[47] Astó E Méndez I Rodríguez-Prado M Cuñé J Espadaler J Farran-Codina A. Effect of the degree of polymerization of fructans on *ex vivo* fermented human gut microbiome. Nutrients. (2019) 11:1293. doi: 10.3390/nu11061293 31181638 PMC6627432

[48] Vacca M Celano G Calabrese FM Portincasa P Gobbetti M De Angelis M. The controversial role of human gut lachnospiraceae. Microorganisms. (2020) 8:573. doi: 10.3390/microorganisms8040573 32326636 PMC7232163

[49] Chua HH Chou HC Tung YL Chiang BL Liao CC Liu HH . Intestinal dysbiosis featuring abundance of *Ruminococcus gnavus* associates with allergic diseases in infants. Gastroenterology. (2018) 154:154–67. doi: 10.1053/j.gastro.2017.09.006 28912020

[50] Tulic MK Vivinus-Nébot M Rekima A Rabelo Medeiros S Bonnart C Shi H . Presence of commensal house dust mite allergen in human gastrointestinal tract: a potential contributor to intestinal barrier dysfunction. Gut. (2016) 65:757–66. doi: 10.1136/gutjnl-2015-310523 26646935

[51] Hosmer J McEwan AG Kappler U. Bacterial acetate metabolism and its influence on human epithelia. Emerg Top Life Sci. (2023) 8:1. 36945843 10.1042/ETLS20220092 PMC10903459

[52] Perdijk O Butler A Macowan M Chatzis R Bulanda E Grant RD . Antibiotic-driven dysbiosis in early life disrupts indole-3-propionic acid production and exacerbates allergic airway inflammation in adulthood. Immunity. (2024) 57:1939–54.e7. doi: 10.1016/j.immuni.2024.06.010 39013465

[53] Kelly RS Sordillo JE Lasky-Su J Dahlin A Perng W Rifas-Shiman SL . Plasma metabolite profiles in children with current asthma. Clin Exp Allergy. (2018) 48:1297. doi: 10.1111/cea.13183 29808611 PMC6160355

[54] Wypych TP Pattaroni C Perdijk O Yap C Trompette A Anderson D . Microbial metabolism of l-tyrosine protects against allergic airway inflammation. Nat Immunol. (2021) 22:279–86. doi: 10.1038/s41590-020-00856-3 33495652

[55] Morillas-Armenta J Macías-Camero A Rzetecka N Zubeldia-Varela E Barker-Tejeda TC Escribese MM . Microbial metabolites in allergic diseases: beyond short-chain fatty acids. Curr Allergy Asthma Rep. (2025) 25:53. doi: 10.1007/s11882-025-01231-8 41214356

[56] Arifuzzaman M Won TH Li TT Yano H Digumarthi S Heras AF . Inulin fibre promotes microbiota-derived bile acids and type 2 inflammation. Nature. (2022) 611:578–84. doi: 10.1038/s41586-022-05380-y 36323778 PMC10576985

[57] Logtenberg MJ Akkerman R An R Hermes GDA de Haan BJ Faas MM . Fermentation of chicory fructo-oligosaccharides and native inulin by infant fecal microbiota attenuates pro-inflammatory responses in immature dendritic cells in an infant-age-dependent and fructan-specific way. Mol Nutr Food Res. (2020) 64:68. doi: 10.1002/mnfr.202000068 32420676 PMC7378940

[58] Mao B Gu J Li D Cui S Zhao J Zhang H . Effects of different doses of Fructooligosaccharides (FOS) on the composition of mice fecal microbiota, especially the bifidobacterium composition. Nutrients. (2018) 10:1105. doi: 10.3390/nu10081105 30115879 PMC6115998

[59] Prattico C Gonzalez E Dridi L Jazestani S Low KE Abbott DW . Identification of novel fructo-oligosaccharide bacterial consumers by pulse metatranscriptomics in a human stool sample. mSphere. (2025) 10:e0066824. doi: 10.1128/msphere.00668-24 39699190 PMC11774028

[60] Fuseini H Newcomb DC. Mechanisms driving gender differences in asthma. Curr Allergy Asthma Rep. (2017) 17:19. doi: 10.1007/s11882-017-0686-1 28332107 PMC5629917

[61] McGee JS Huttenhower C. Of mice and men and women: sexual dimorphism of the gut microbiome. Int J Womens Dermatol. (2021) 7(5 Part A):533. doi: 10.1016/j.ijwd.2021.10.007 35005176 PMC8721129

[62] Hugenholtz F de Vos WM. Mouse models for human intestinal microbiota research: a critical evaluation. Cell Mol Life Sci. (2017) 75:149. doi: 10.1007/s00018-017-2693-8 29124307 PMC5752736

[63] Dhariwal A Chong J Habib S King IL Agellon LB Xia J. MicrobiomeAnalyst: a web-based tool for comprehensive statistical, visual and meta-analysis of microbiome data. Nucleic Acids Res. (2017) 45:W180. doi: 10.1093/nar/gkx295 28449106 PMC5570177

