# Supplementary material for: Dietary fructo-oligosaccharides dose-dependently modulate the microbiome and suppress type 2 lung inflammation in a murine model of house dust mite-induced allergic asthma
Source: Front Nutr. 2026 Mar 23;13:1705988. doi: 10.3389/fnut.2026.1705988 (PMC13051382; doi:10.3389/fnut.2026.1705988)
Supplement: Supplementary file 1 [file Data_Sheet_1.docx]

# Supplemental information

**Supplemental table 1. Nutritional composition of the dietary interventions, per intervention.**


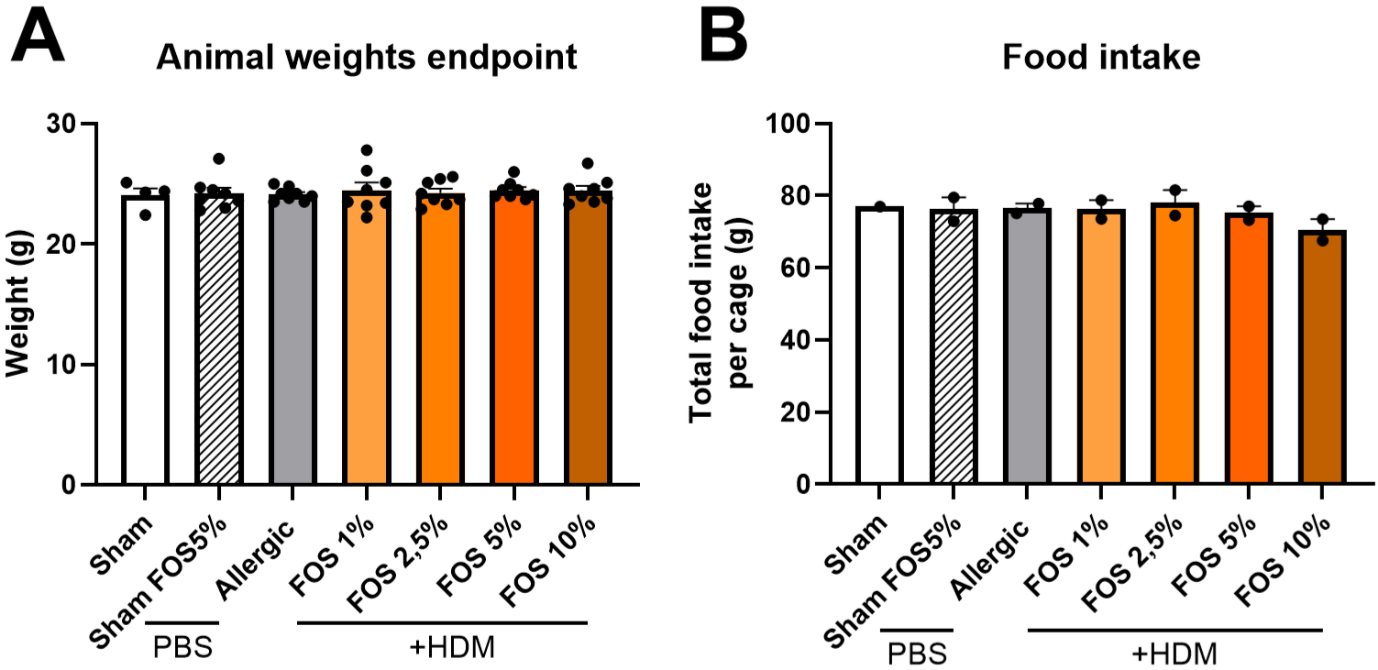


**Supplemental figure 1. General data on animals.** A) Body weight of mice at the last day of the experiment. B) Total amount of food eaten by an individual mouse during the course of the experiment. Amount is calculated by dividing the total amount of food eaten in a cage by the number of mice in that cage. Differences were tested using a One-Way ANOVA with Tukey’s multiple comparisons test, comparing all groups with each other. For A, N = 8 (sham N =4), for B), N = 2 (sham N=1). Results are shown as mean ± SEM.


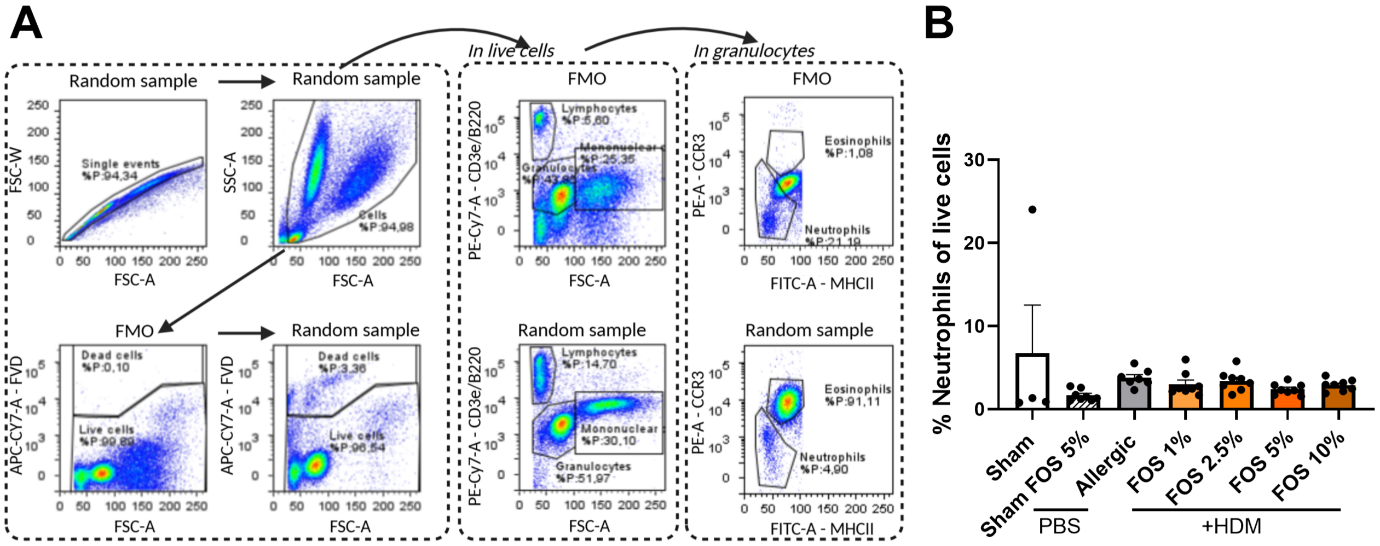


**Supplemental figure 2.** **Differentiation of inflammatory cells in the broncho-alveolar lavage fluid (BALF).** Representative gating strategy, including corresponding FMOs, for flow cytometric differentiation of cells obtained from BALF. The left block describes the gating of the single cell and live cell fraction defined as fixable-viability-dye (FVD) negative. The middle block describes the differentiation between lymphocytes (FSC_low_-CD3e/B220_high_), granulocytes (FSC_low_-CD3e/B220_low_) and mononuclear cells (FSC_high_) in the live cell fraction. The right block describes the eosinophil (CCR3+) and neutrophil (CCR3-) populations in the granulocyte gate. B) Percentage of neutrophils in BALF as determined by flow cytometry. The experimental model (Sham vs Allergic), and sham-5% FOS effect comparisons were analyzed using a Welch’s t test. Effect of the diets on HDM allergy (Allergic (HDM)-control diet vs Allergic (HDM)- FOS diet groups) were tested using One-Way ANOVA followed by post hoc Dunnett’s multiple comparisons test. N = 7-8 (sham N =4). Results are shown as mean ± SEM.


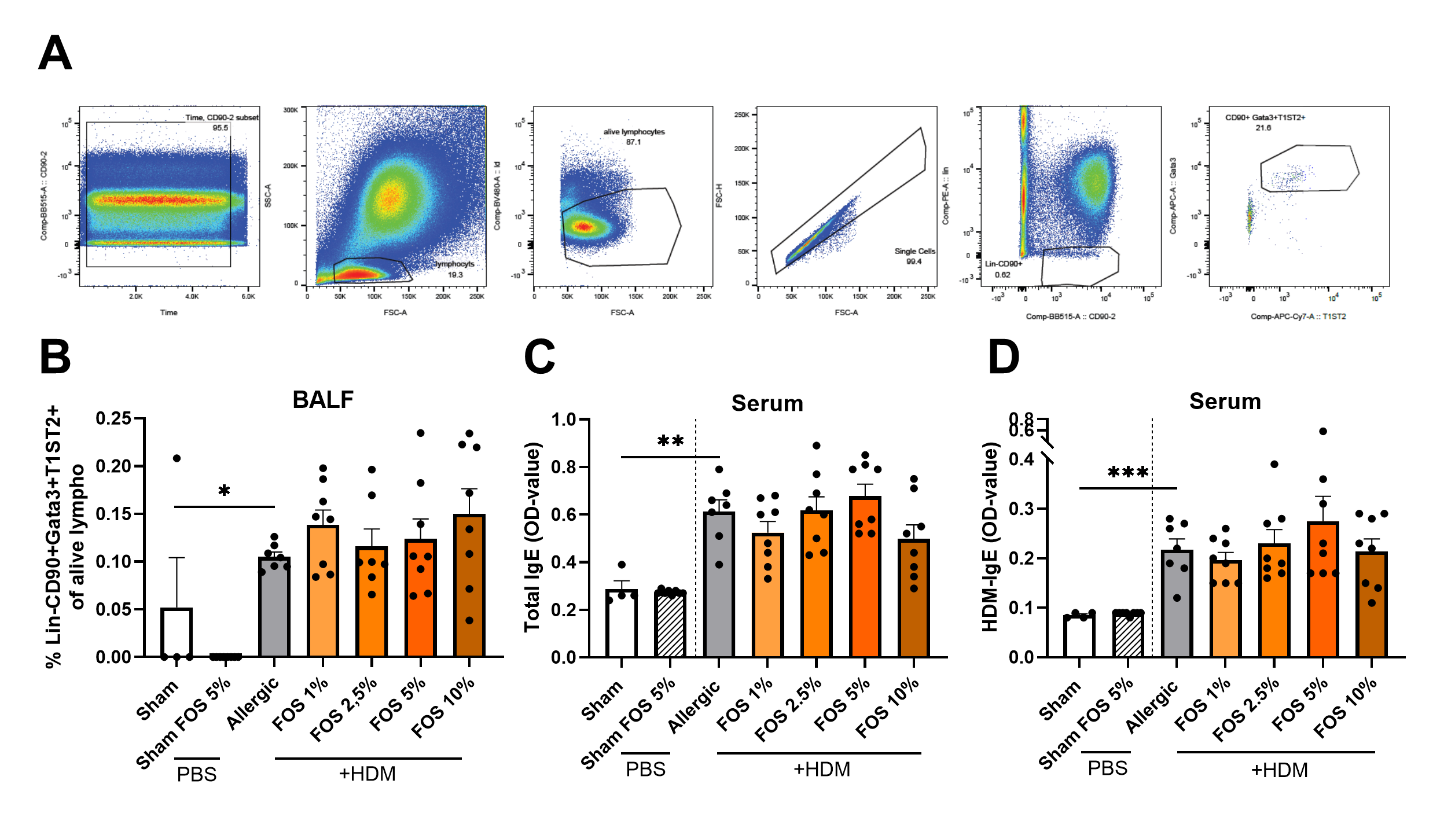


**Supplemental figure 3. Effects of FOS on ILC2 frequency in the broncho-alveolar lavage fluid (BALF) and serum antibodies.** ILC2 frequency was determined by flow cytometry. A) Representative gating strategy for identification of ILC2 cells. B) ILC2 levels (Lin-CD90+GATA3+T1ST2+) expressed as % of total lymphocytes. Serum levels of C) Total IgE and D) HDM-IgE were determined by ELISA. Values are expressed as the read-out value (optical density). The model (Sham vs Allergic) was validated, and sham FOS effect (Sham vs Sham FOS 5%) was analyzed using an unpaired t-test. Effect of the diets on allergy (Allergic (HDM)-control diet vs Allergic (HDM)- FOS diet groups) were tested using One-Way ANOVA followed by post hoc Dunnett’s multiple comparisons test. N = 7-8 (sham N =4). Results are shown as mean ± SEM (*P<0.05, **P<0.01, *** P < 0.001).


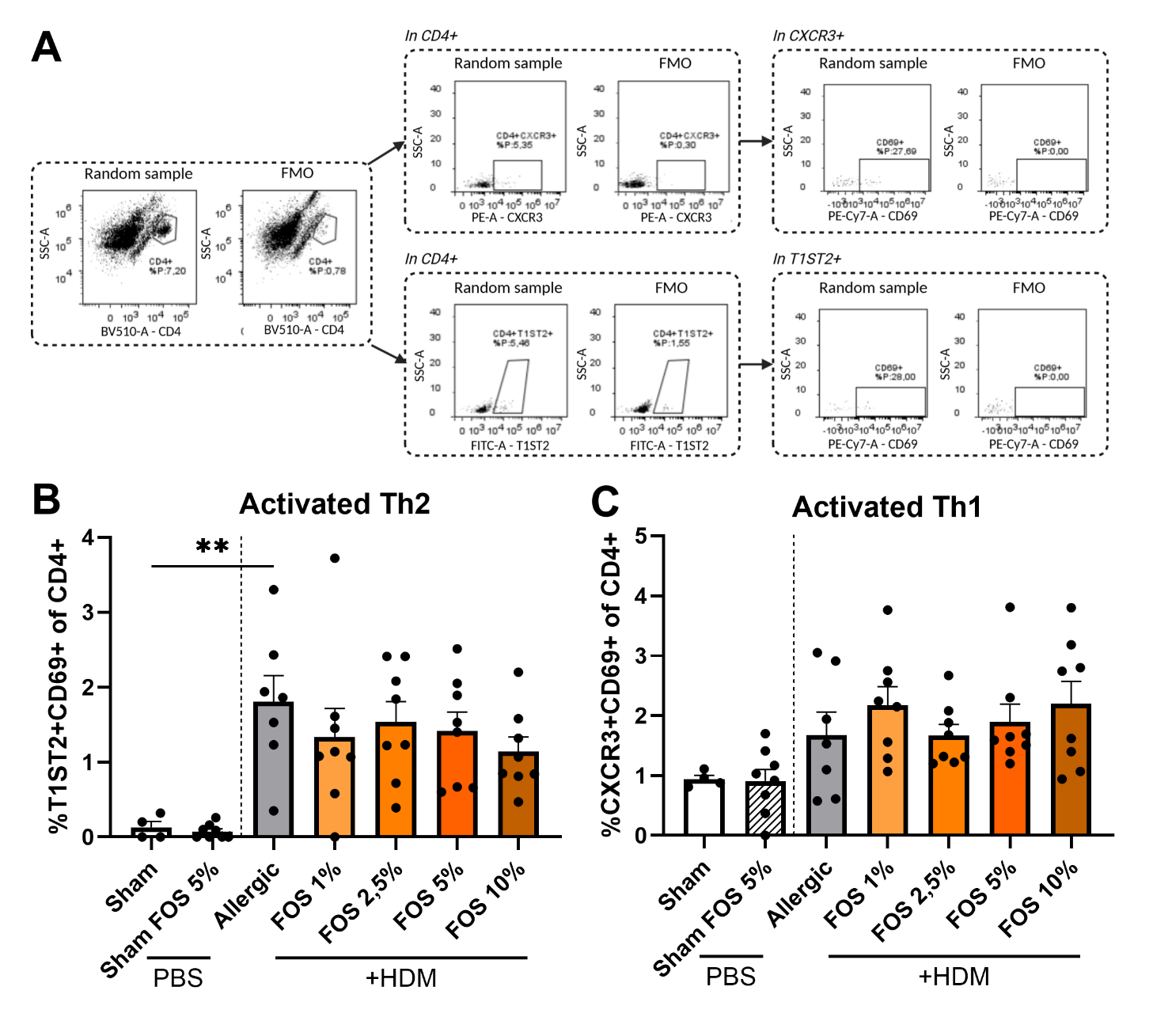


**Supplemental figure 4.** **Differentiation of Th2 and Th1 cells in the lung.** A) Representative gating strategy, including corresponding FMOs, for flow cytometric differentiation of cells obtained from lungs. The left block describes the gating of the CD4+ fraction within the live cells gate. The next upper blocks describes the CXCR3+ (Th1) population within the CD4+ gate, and consequently the CD69+ (activated) population within the CXCR3+ gate. The lower blocks describes the T1ST2+ (Th2) population within the CD4+ gate, and consequently the CD69+ (activated) population within the T1ST2+ gate. Frequency of B) Activated Th2 cells and C) Activated Th1 cells. The experimental model (Sham vs Allergic), and sham vs 5% FOS effect comparisons were analyzed using an unpaired t-test or Welch’s t-test. Effect of the diets on HDM allergy (Allergic (HDM)-control diet vs Allergic (HDM) -FOS diet groups) were tested using One-Way ANOVA followed by post hoc Dunnett’s multiple comparisons test. N = 7-8 (sham N =4). Results are shown as mean ± SEM (**p<0.01).


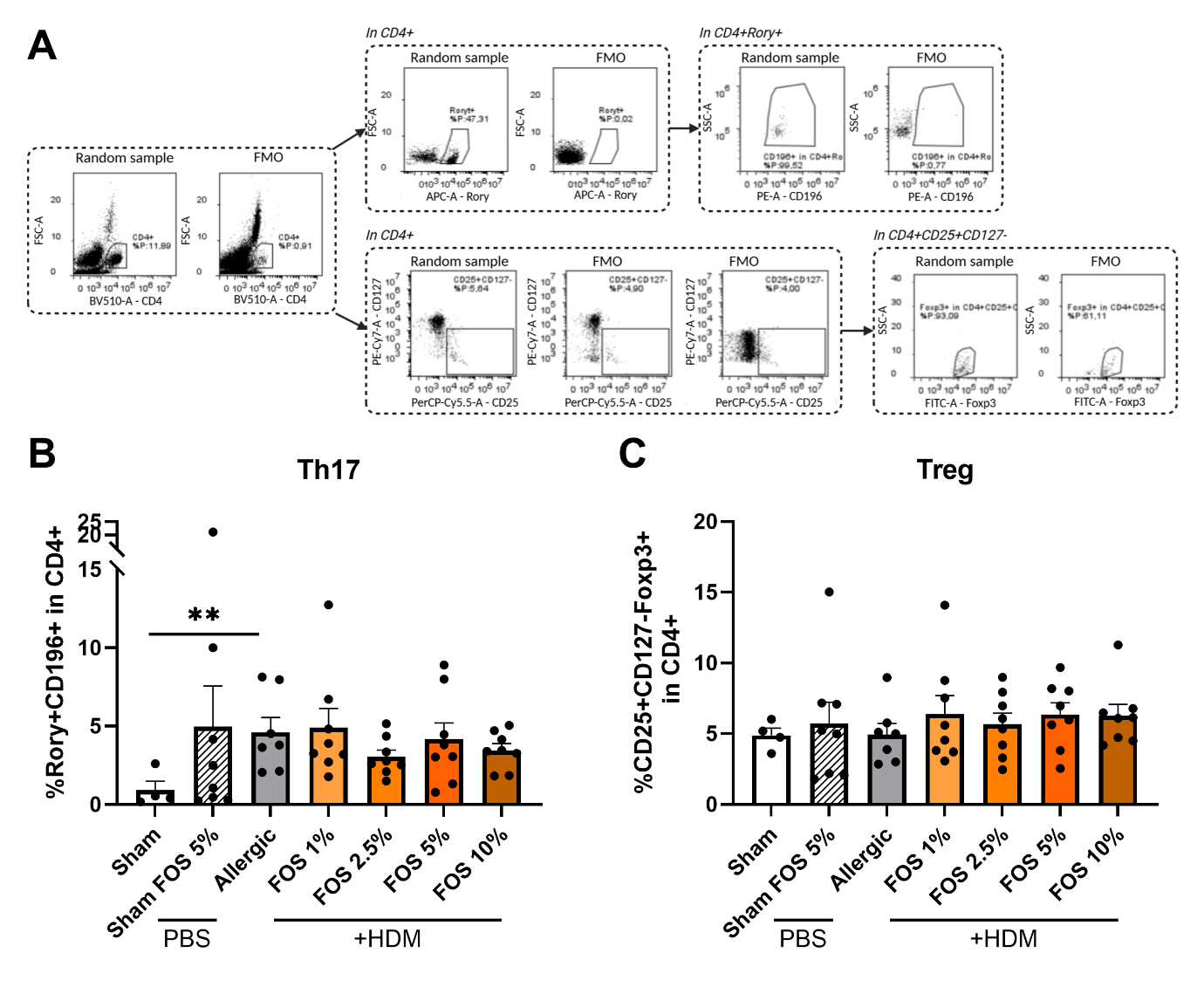


**Supplemental figure 5. Differentiation of Th17 and Treg cells in the lung.** A) Representative gating strategy, including corresponding FMOs, for flow cytometric differentiation of cells obtained from lungs. The left block describes the gating of the CD4+ fraction within the live cells gate. The next upper blocks describes the Rorγ+ population within the CD4+ gate, and consequently the CD196+ population within the CD4+Rorγ+ gate (Th17). The lower blocks describes the CD25+CD127-population within the CD4+ gate, and consequently the Foxp3+ population within the CD4+CD25+CD127- gate (Treg). Frequency of B) Th17 cells and C) Treg cells. The experimental model (Sham vs Allergic), and sham vs 5% FOS effect comparisons were analyzed using an unpaired t-test or Welch’s t-test. Effect of the diets on HDM allergy (Allergic (HDM)-control diet vs Allergic (HDM) -FOS diet groups) were tested using One-Way ANOVA followed by post hoc Dunnett’s multiple comparisons test. N = 7-8 (sham N =4). Results are shown as mean ± SEM (**p<0.01).


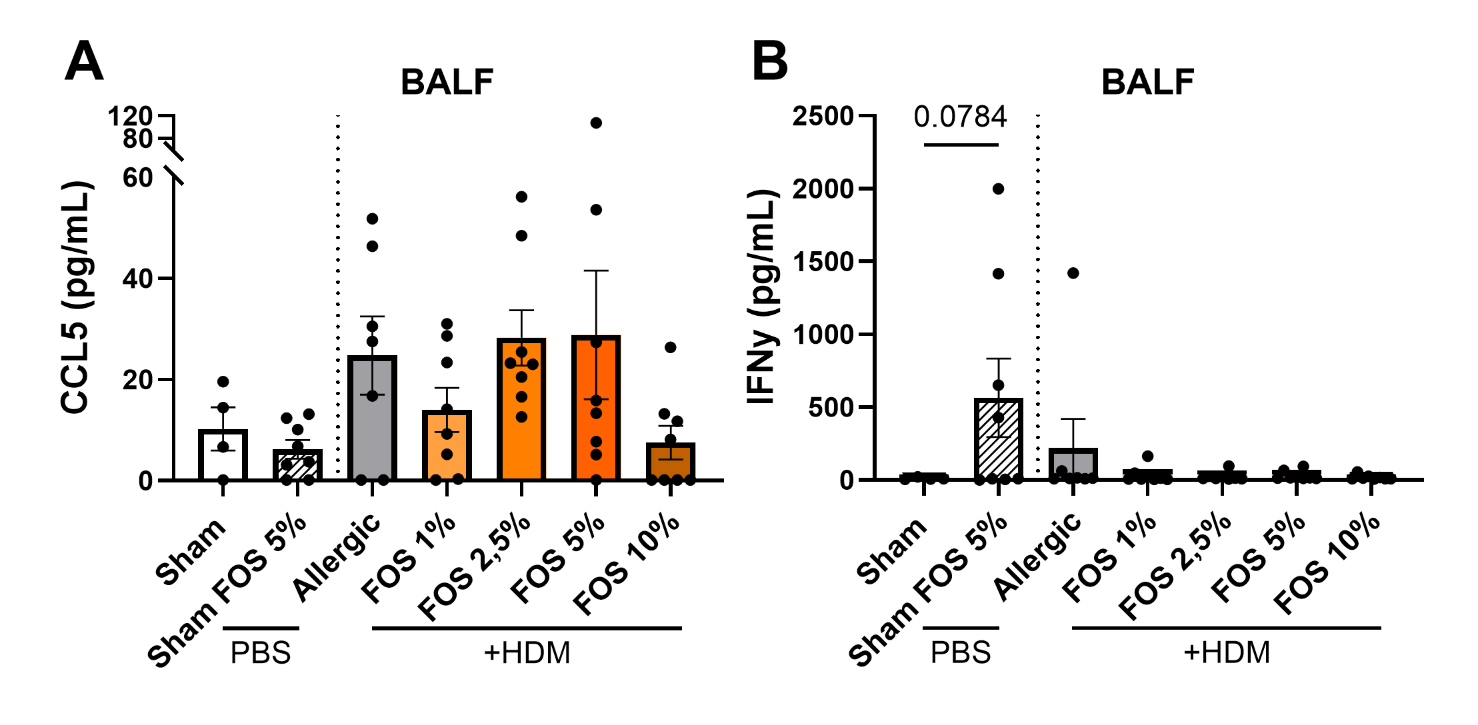


**Supplemental figure 6.** **Effects of FOS on cytokine levels in the BALF of HDM-allergic mice**. Concentrations of A) CCL5 and B) IFNγ were determined. The experimental model (Sham vs Allergic), and sham vs 5% FOS comparisons were analyzed using an unpaired t-test or Welch’s t-test. Effect of the diets on HDM allergy (Allergic (HDM)-control diet vs Allergic (HDM) -FOS diet groups) were tested using One-Way ANOVA followed by post hoc Dunnett’s multiple comparisons test. N = 7-8 (sham N =4). Results are shown as mean ± SEM.


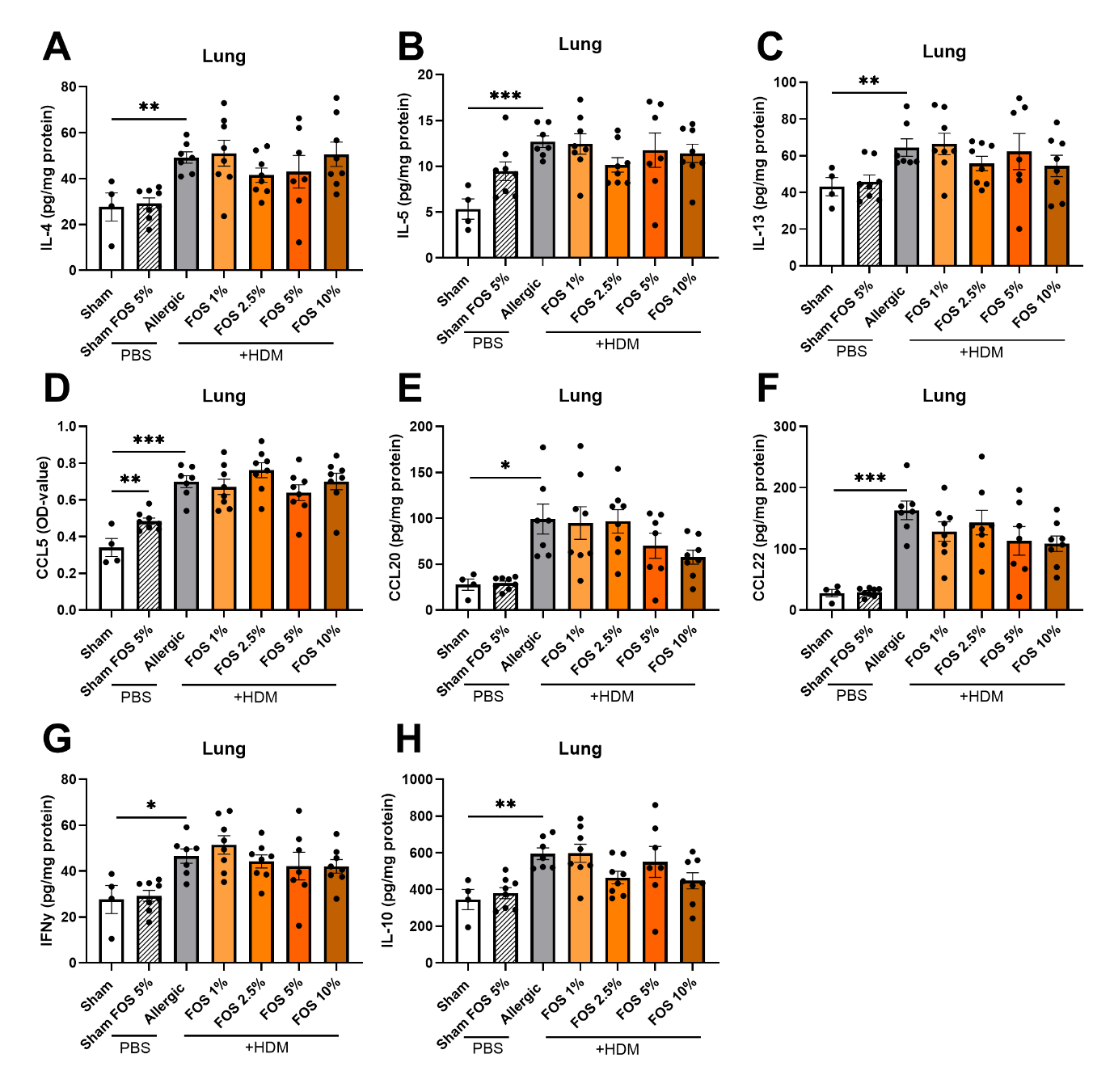


**Supplemental figure 7. Effects of FOS on cytokine and chemokine levels in the lung homogenates.** Cytokine and chemokine levels were determined using ELISA. Measured levels were corrected for the total protein concentration in the samples. A) IL-4, B) IL-5 and C) IL-13 were included as Th2-associated cytokines. D) CCL5, E) CCL20 and F) CCL22 were included as allergic asthma related chemokines. G) IFNγ and H) IL-10 were included as Th1-associated cytokines. The experimental model (Sham vs Allergic), and sham vs 5% FOS comparisons were analyzed using an unpaired t-test. Effect of the diets on HDM allergy (Allergic (HDM)-control diet vs Allergic (HDM) -FOS diet groups) were tested using One-Way ANOVA followed by post hoc Dunnett’s multiple comparisons test. N = 7-8 (sham N =4). Results are shown as mean ± SEM. (*p<0.05, **p<0.01, ***p<0.01).


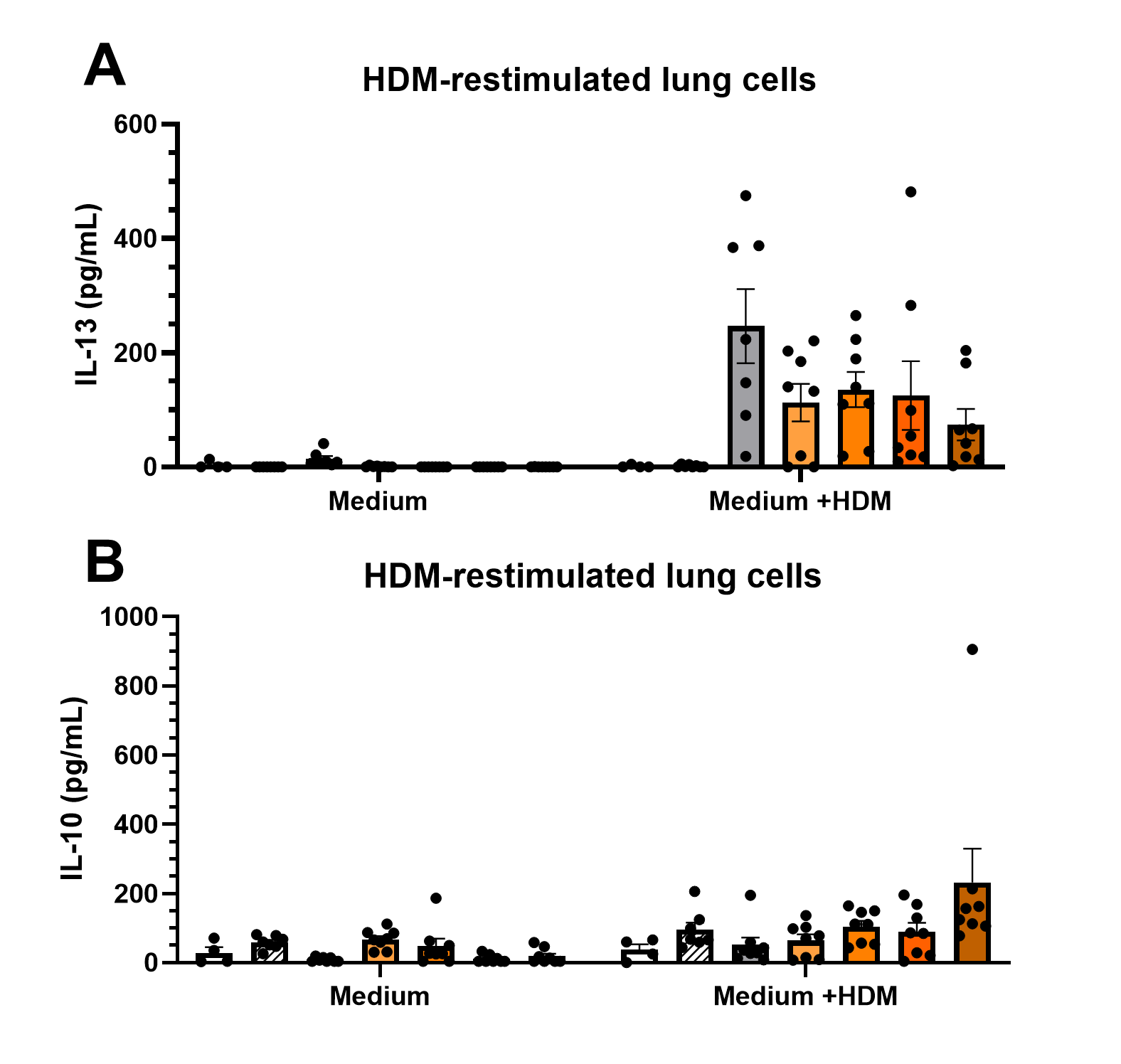


**Supplemental figure 8. Ex vivo restimulation of lung cells of HDM-allergic mice. Comparing medium exposed cells with cells exposed to medium with HDM.** Lung cells were isolated and ex vivo cultured and restimulated with HDM. Cytokine concentrations of A) IL-13 and B) IL-10 were measured in the supernatant of the cell culture after 6 days of incubation. N = 7-8 (sham N =4). Results are shown as mean ± SEM.


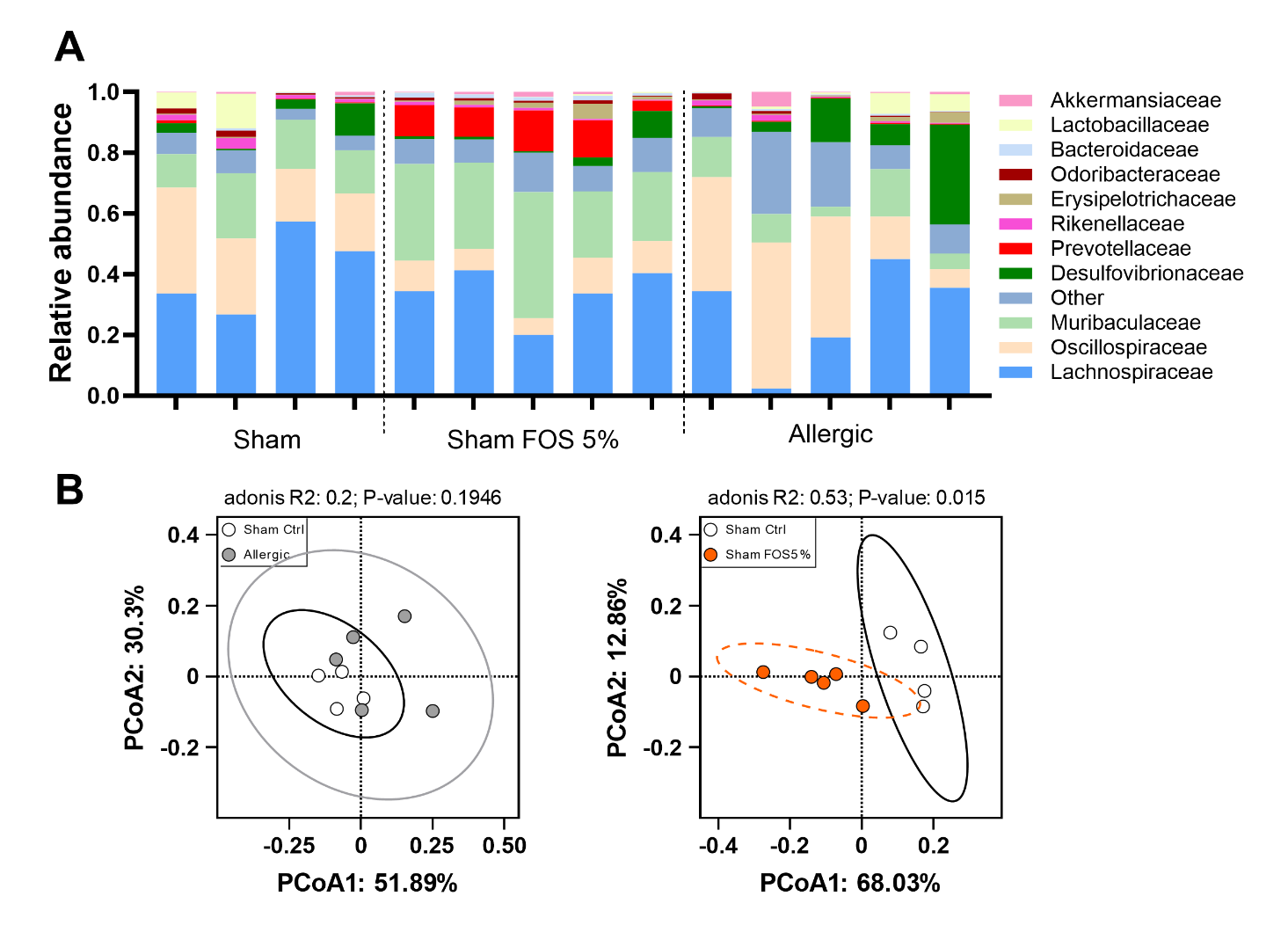


**Supplemental figure 9.** **Microbiome composition of control groups.** Faecal samples of a random selection of mice from each experimental group were analyzed using 16S rRNA-gene sequencing (Sham N=4, Other N=5). A) Microbiome profiles of sham mice, sham mice receiving 5% FOS and allergic mice. Relative abundance of the top 10 bacterial families found in the faecal samples are displayed. B) Principle Coordinates Analysis for beta diversity between sham and allergic group, and between sham and sham FOS 5% group, including Adonis value.


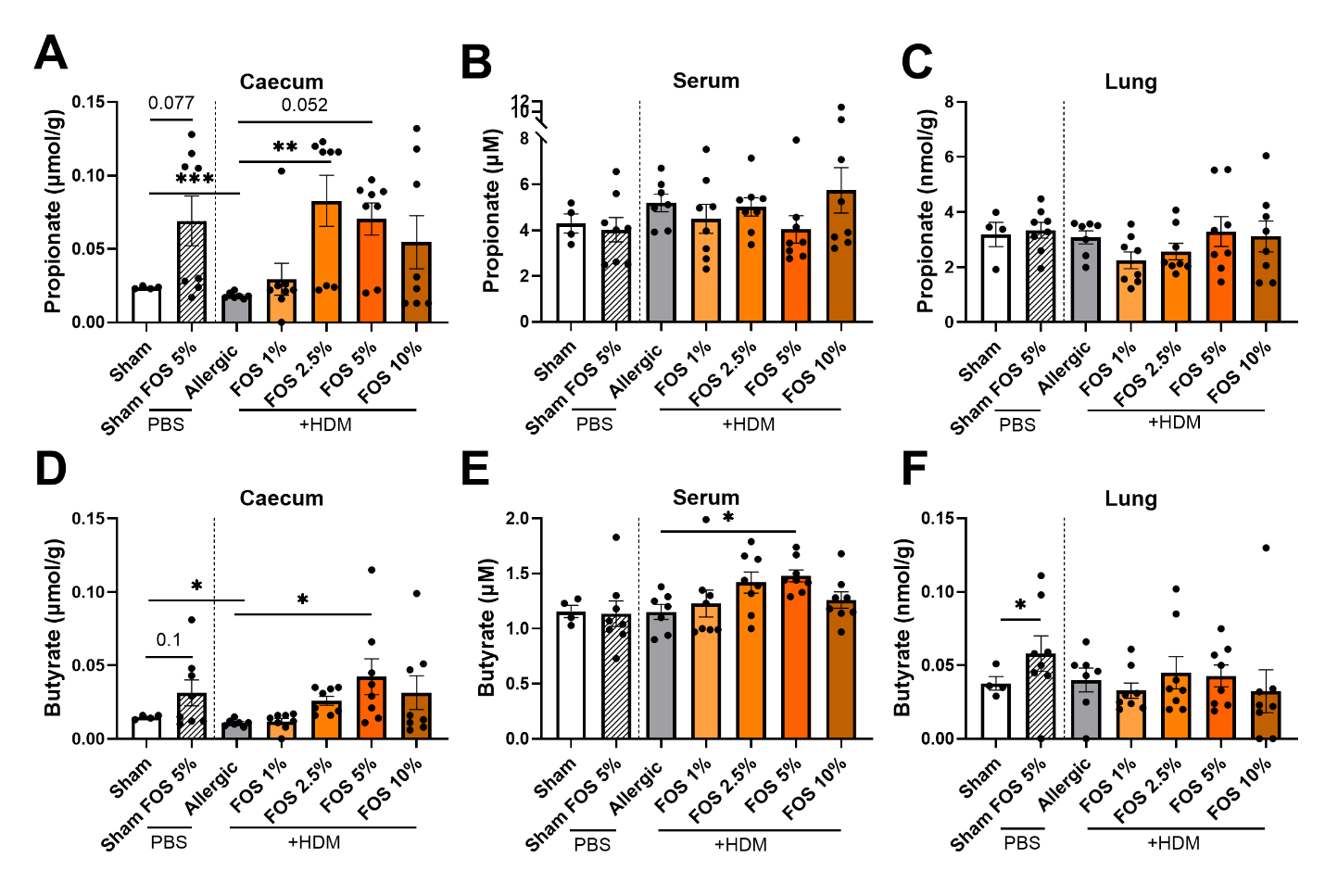


**Supplemental figure 10. Propionate and butyrate levels in caecum content, serum and lung.** Propionate and butyrate levels were quantified using LC-MS/MS, in A&D) caecum content, B&E) serum and C&F) lungs (Sham N=4, Other N=7-8). The experimental model (Sham vs Allergic) and sham-5% FOS effect comparisons were analyzed using an unpaired t-test or Welch’s t test. Effect of the diets on HDM allergy (Allergic (HDM)-control diet vs Allergic (HDM)- FOS diet groups) were tested using One-Way ANOVA followed by post hoc Dunnett’s multiple comparisons test or Kruskal-Wallis with post hoc Dunn’s multiple comparisons test. Results are shown as mean ± SEM (*P<0.05, **P<0.01, ***P<0.001).
